# Pharmacological targeting of P300/CBP reveals EWS::FLI1-mediated senescence evasion in Ewing sarcoma

**DOI:** 10.1186/s12943-024-02115-7

**Published:** 2024-10-05

**Authors:** Erdong Wei, Ana Mitanoska, Quinn O’Brien, Kendall Porter, MacKenzie Molina, Haseeb Ahsan, Usuk Jung, Lauren Mills, Michael Kyba, Darko Bosnakovski

**Affiliations:** 1 Department of Pediatrics, University of Minnesota, 2231 6th St. SE, Minneapolis, MN 55455 USA; 2 Lillehei Heart Institute, University of Minnesota, Minneapolis, MN 55455 USA; 3 Minneapolis, USA

**Keywords:** Ewing sarcoma, EWS::FLI1, Lamin B1, P300/CBP, Senescence, Senolytics, Pharmacological targeting

## Abstract

Ewing sarcoma (ES) poses a significant therapeutic challenge due to the difficulty in targeting its main oncodriver, EWS::FLI1. We show that pharmacological targeting of the EWS::FLI1 transcriptional complex via inhibition of P300/CBP drives a global transcriptional outcome similar to direct knockdown of EWS::FLI1, and furthermore yields prognostic risk factors for ES patient outcome. We find that EWS::FLI1 upregulates LMNB1 via repetitive GGAA motif recognition and acetylation codes in ES cells and EWS::FLI1-permissive mesenchymal stem cells, which when reversed by P300 inhibition leads to senescence of ES cells. P300-inhibited senescent ES cells can then be eliminated by senolytics targeting the PI3K signaling pathway. The vulnerability of ES cells to this combination therapy suggests an appealing synergistic strategy for future therapeutic exploration.

**Supplementary Information:**

The online version contains supplementary material available at 10.1186/s12943-024-02115-7.

## One sentence summary

Pharmacological inhibition of P300/CBP disrupts the EWS::FLI1 transcriptional complex in Ewing sarcoma, leading to senescence and vulnerability to senolytics, providing a promising therapeutic strategy for this challenging cancer.

## Introduction

Ewing sarcoma (ES), the second most common form of pediatric bone cancer, primarily affects individuals at a median age of 15 years and occurs with an incidence of approximately three cases per million annually [1, 2]. Over the last fifty years, improvements in diagnosis, surgery, chemotherapy, and radiation have increased survival rates to nearly 70% for localized ES, yet the prognosis for patients with metastatic or recurrent forms remains challenging, with a five-year survival rate still below 25% [3, 4]. This disparity underscores an urgent need for new therapeutic targets and strategies.

ES is defined by chromosomal translocations that form fusion genes encoding aberrant transcription factors crucial for pathogenesis. The predominant translocation, t(11;22)(q24;q12), present in about 85% of cases, leads to the formation of EWS::FLI1 [5]. Acting as a transcription factor, the EWS::FLI1 fusion protein alters cellular functions and promotes the expression of key oncogenes such as CMYC, ID2, and CCND1, as well as genes associated with super enhancer activities like MEIS1 and APCDD1 [6, 7]. The preferential binding of EWS::FLI1 to GGAA microsatellites creates enhancers that amplify expression of the oncogene, promoting tumor aggression and treatment resistance [8].

However, the structural complexity of EWS::FLI1 and the absence of a defined active site make direct targeting a challenge [9, 10]. Research efforts are thus increasingly focused on understanding and disrupting the regulatory network of EWS::FLI1 as a strategy to counteract its oncogenic effects [2, 6, 11].

The oncogenic potential of EWS::FLI1 is highly dependent on the cellular context, as evidenced by its varying effects across different cell types [12]. In permissive environments like mesenchymal stem (MSC) and neuroectodermal cells, the EWS::FLI1 fusion protein not only drives ES-like transformation and tumorigenesis by promoting cellular proliferation [12], but also significantly contributes to immune evasion [13]. This oncogenic protein modulates immune-related gene expression, alters cytokine profiles to favor immunosuppression, interferes with effective antigen presentation by downregulating MHC class I molecules, and manipulates the tumor microenvironment to encourage the recruitment of immunosuppressive cells like M2 macrophages [13]. EWS::FLI1 toxicity is apparent when expressed in non-permissive cells, as it triggers cellular stress responses that result in cell cycle arrest, apoptosis, or senescence [14].

P300 (E1A Binding Protein P300) and CBP (CREB Binding Protein) are critical histone acetyltransferases that mark promoters and enhancers for gene activation via acetylation of histone H3 at lysine 27 (acH3K27) [15]. This modification is essential for cancer progression in various types such as liver cancer, prostate cancer, melanoma, renal carcinoma, leukemia, lymphoma, and lung cancer [15]. Acetylation by P300/CBP enhances cell proliferation, survival, and metastasis, contributing to therapy resistance and immune evasion mechanisms [15]. There is a crucial partnership in ES, where P300/CBP is dynamically localized to interact with the activation domain of the EWS::FLI1 fusion protein. This results in enhanced oncogenic capacity through chromatin relaxation and remodeling, and subsequent tumor progression [6, 8, 16, 17]. Given the challenges of directly targeting EWS::FLI1, our study focuses on P300/CBP as alternative therapeutic targets, aiming to disrupt this crucial interaction and thereby reduce oncogenic activity.

Despite their crucial role in cellular processes, the specific function of P300/CBP in ES remains uncharted [15]. This study underscores the dependence of the EWS::FLI1 fusion protein on P300/CBP to govern vital downstream targets crucial for ES cell growth and malignancy. We unravel the intriguing regulatory mechanism by which EWS::FLI1 prevents ES cancer cells and permissive cells from entering senescence, acting concurrently as a transcriptional activator of LMNB1 and repressor of P15. Moreover, we demonstrate pharmacological targeting of the EWS::FLI1/P300/CBP axis holds promise as a therapeutic approach for ES.

## Results

### siRNA-mediated knockdown of P300/CBP affects ES cell viability

**Fig. 1:**
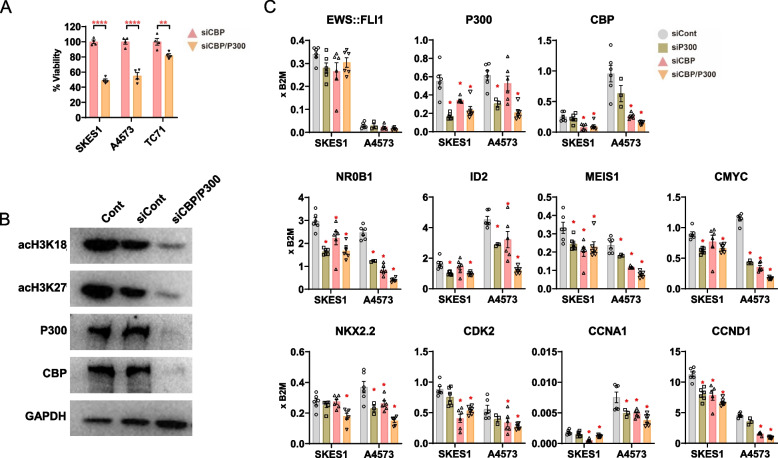
siRNA-mediated knockdown of P300/CBP unveils a pivotal role in ES. **A** Bar graphs show the significant reduction in cell viability across SKES1, A4573, and TC71 ES cell lines after siRNA-mediated knockdown of P300/CBP at 72 hours. The data represent mean ± SEM; **p<0.001, ****p<0.0001 by two-way ANOVA, *n*=4. Results are presented as percentage viability, normalized to the control sample. **B** Western blot analysis confirms the knockdown of P300/CBP at the protein level and reveals decreased levels of acetylated histone markers (acH3K18 and acH3K27) after 48 hours of siRNA treatment. “Cont” refers to untreated cells, “siCont” indicates cells treated with scramble siRNA, and “siCBP/P300” refers to cells treated with siRNA targeting both CBP and P300. **C** RT-qPCR analysis at 48 hours following P300/CBP knockdown shows decreased expression of EWS::FLI1 regulated genes (NR0B1, MEIS1, c-MYC, ID2) in SKES1 and A4573 cell lines. Silencing P300 or CBP individually led to a reduction in the target genes, with a more pronounced effect observed upon concurrent silencing of both. Data are presented as mean ± SEM; *p<0.05, by two-way ANOVA. Results are presented as relative expression to B2M (*n*=6)

While P300/CBP is recognized as a promoter of cancer cell proliferation [15, 18], its impact on ES viability remains uncertain. The influence of P300/CBP on the survival of three ES cell lines, SKES1, A4573, and TC71, was evaluated through siRNA-mediated knockdown. P300/CBP-targeted siRNA decreased cell viability across all examined ES cell lines, underscoring the vital role these histone acetyltransferases play in cell survival (Fig. 1A). The efficiency of siRNA-mediated knockdown was confirmed by assessing P300 and CBP protein levels via Western blot (Fig. 1B). Knockdown of P300/CBP led to a reduction in acetylated H3K18 and H3K27, indicating a shift toward a transcriptionally repressive chromatin state. Knockdown of either P300 or CBP reduced the expression of well-known targets of EWS::FLI1, such as NR0B1, MEIS1, and C-MYC levels in SKES1 and A4573 ES cell lines. However, silencing both P300 and CBP together had a more pronounced effect, resulting in heightened downregulation across a plethora of targets (Fig. 1C). Thus, knockdown of P300/CBP by RNAi emphasizes its indispensable role within the EWS::FLI1 transcriptional axis and in maintaining ES cell viability.

### Pharmacological inhibition P300/CBP inactivates EWS::FLI1 oncogenic activity

**Fig. 2:**
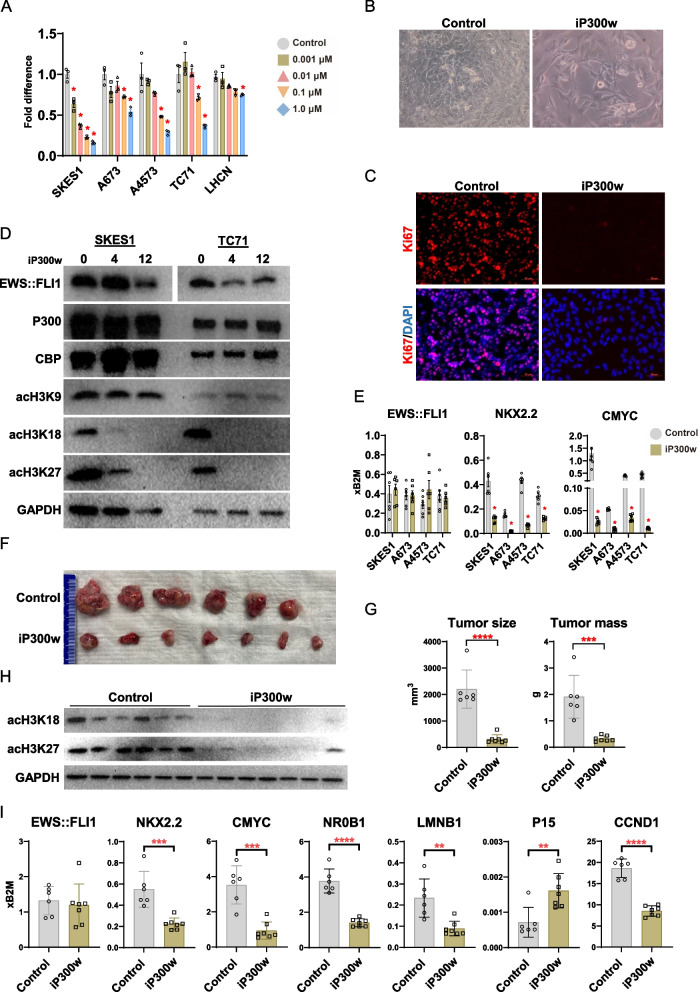
Successful pharmacological targeting of ES using iP300w. **A** Bar graphs show the dose-dependent inhibition of viability in ES cell lines (SKES1, A673, A4573, and TC71) compared to normal human myoblast cells (LHCN) following iP300w treatment for 48 hours. The ATP assay data are represented as mean ± SEM; *p<*0.05, by two-way ANOVA. Results are presented as fold difference compared to control (untreated group at 48 hours) (*n*=3). **B** SKES1 cell morphology after 48 hours of treatment with 1µM iP300w. Scale bar 100µm. **C** Immunofluorescence for Ki67 (red) expression in SKES1 cells indicates cell-cycle arrest after 48 hours of iP300w treatment (1 µM). DAPI (blue) was used to stain the nuclei. **D** Western blot analysis reveals for EWS::FLI1, P300, CBP, acH3K9, acH3K18 and acH3K27, in SKES1 and TC71 cell lines treated with 1 µM iP300w for 4 and 12 hours. **E** Bar graphs show RT-qPCR results illustrating unchanged EWS::FLI1 expression following P300/CBP inhibition, with a significant reduction in EWS::FLI1 target gene expression (NKX2.2 and CMYC) at 24 hours post-treatment. Data were normalized to B2M. The data represent the mean ± SEM, *p<* 0.05, by t-test. Results are presented as fold differences compared to the control (untreated group at 24 hours) (*n* = 6). **F** Gross morphology of tumors from treated (iP300w, 5.6 mg/kg daily) and control mice at the terminal point of the experiment (day 14). **G** The graphs display tumor volume and weight measurements, demonstrating significant reductions in tumor size and mass following treatment. Data are presented as mean ± SEM; ***p< 0.001, ****p< 0.0001, by t-test (control, *n*=6; treatment, *n*=7). **H** Western blot for acetylated H3K18 and H3K27 in tumor samples presented in “F”. **I** RT-qPCR analysis of EWS::FLI1, its target genes, senescence-related genes, and cell cycle genes in the xenografts from experiment “F”. Data were normalized to B2M and compared to the control group using a t-test. The data represent mean ± SEM; *p< 0.05, **p< 0.01, ***p< 0.001, ****p< 0.0001, (control, *n*=6; treatment, *n*=7)

To pharmacologically emulate the effects of P300/CBP siRNA-mediated knockdown, we treated ES cell lines with our recently developed P300/CBP inhibitor, iP300w [18]. The treatment reduced cell viability in a dose-dependent manner across all tested ES cell lines. All tested ES cell lines exhibited significant sensitivity to iP300w at a concentration of 0.1 μM after 48 h of treatment (Fig. 2A and Supplementary Fig. 1A). In contrast, the viability of the non-sarcoma human myoblast LHCN cell line and the osteosarcoma cell line remained unaffected at this concentration (Fig. 2A and Supplementary Fig. 1A). However, a slight but statistically significant decrease in cell viability was observed in the LHCN and SJSA-1 cell lines at 1.0 μM. The impact of P300/CBP inhibition on cell proliferation and morphology was readily observable under bright-field microscopy. iP300w-treated samples exhibited a marked decrease in cell confluence, along with a noticeable increase in cell size and a more flattened, spread-out morphology. (Fig. 2B). Ki-67 staining demonstrated almost complete suppression of cell proliferation at 48 h of the iP300w treatment (Fig. 2C and Supplementary Fig. 1B).

Next, we investigated the transcriptional and epigenetic effects of iP300w treatment in both SKES1 and TC71 cell lines. Within 4 h, acetylation at H3K18 and H3K27 notably decreased, and was entirely diminished by the 12-h mark (Fig. 2D). As anticipated, there was no change in acetylation levels on H3K9, affirming the iP300w specific inhibition of P300/CBP-mediated H3 acetylation [19]. Concurrently, protein levels of P300 and CBP remained stable, suggesting iP300w modulates the activity of these acetyltransferases without altering their expression (Fig. 2D). Moreover, RT-qPCR analysis at 24 h post-iP300w treatment revealed a significant downregulation of EWS::FLI1 target genes, notably NKX2.2, NR0B1 and C-MYC (Fig. 2E, Supplementary Fig. 1C). Remarkably, the sensitivity of EWS::FLI1 transcriptional activity to treatment was evident within just 4 h (Supplementary Fig. 1D). Notably, EWS::FLI1 protein levels decreased with iP300w treatment while transcription remained unaffected. This suggests that the stability of EWS::FLI1 may be dependent on P300/CBP-mediated acetylation (Fig. 2D, E), similar to the relationship between P300/CBP and other transcription factors like TP53, SOX10, and MCL-1 [20–22].

Finally, we assessed the efficacy of iP300w in suppressing ES tumor formation in a SKES1 xenograft tumor model (Fig. 2F, G and Supplementary Fig. 2A). Mice treated with iP300w exhibited significant reductions in both tumor size and mass compared to control groups. By the end of the experiment (day 14), average tumor volumes were significantly smaller in the treated group (300 mm^3^, 0.3 g) compared to the untreated control group of mice (2200 mm^3^, 2 g) (Fig. 2H, G). Consistent with the in vitro observations, treated tumors showed a notable decrease in H3K18 and H3K27 acetylation, as well as in the expression of EWS::FLI1 target genes (Fig. 2H and I, and Supplementary Fig. 1E). To evaluate the potential side effects of iP300w treatment, we monitored body weight changes (Supplementary Fig. 2B) and assessed serum levels of BUN, creatinine, sodium, potassium, osmolality, ALP, ALT, and glucose (Supplementary Fig. 2C). Among all the parameters, only glucose showed a slight upregulation in the iP300w-treated group. BUN was actually modestly decreased with statistical significance in the treated group. Furthermore, histopathological analysis revealed no noticeable tissue damage or pathological changes in the liver and kidneys (Supplementary Fig. 2D). In summary, iP300w efficiently suppresses EWS::FLI1/P300/CBP oncogenic axis both in vitro and in vivo.

### EWS::FLI1 knockdown and P300/CBP inhibition induce similar transcriptional alterations in ES

**Fig. 3:**
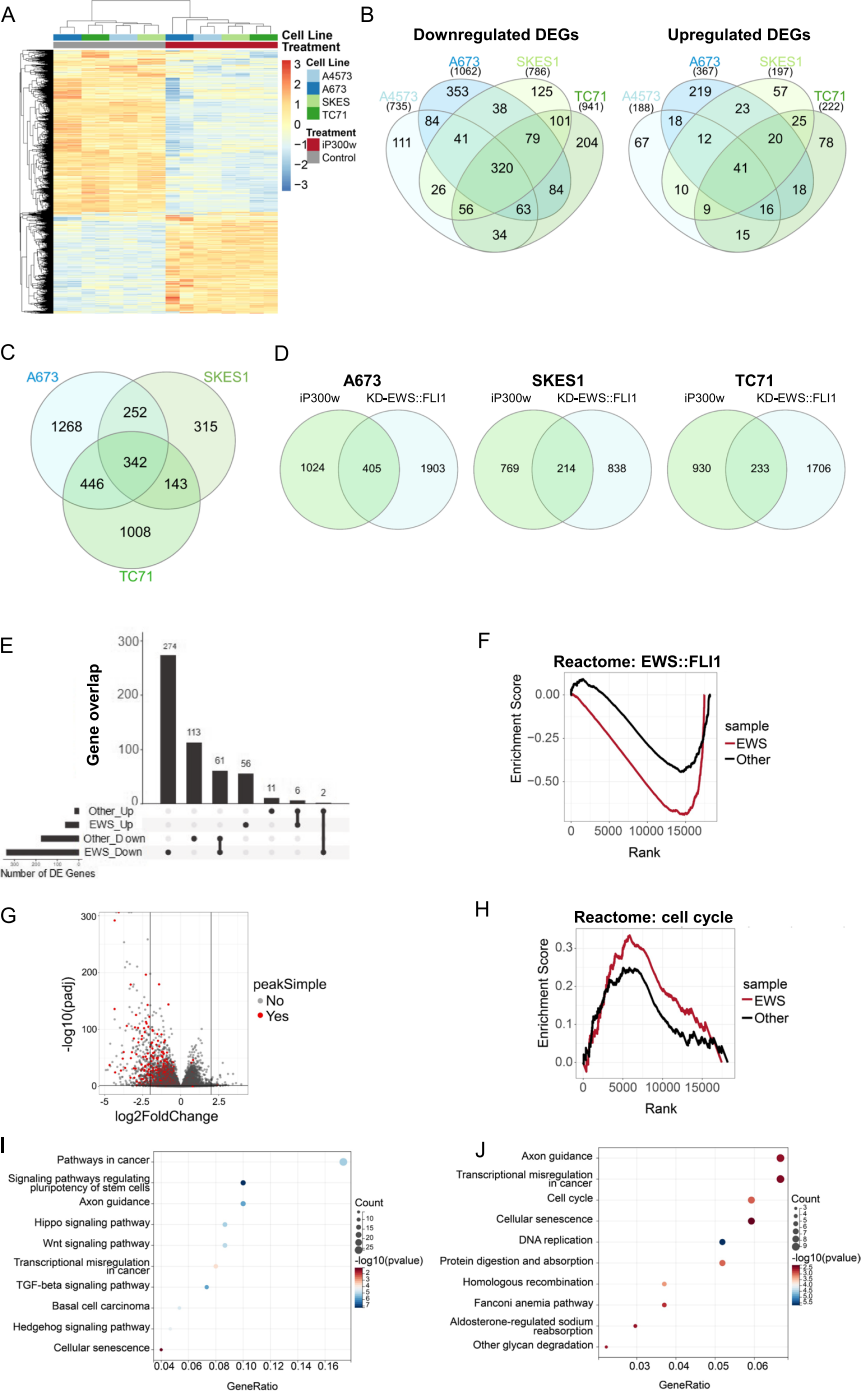
P300/CBP inhibition and EWS::FLI1 knockdown yield comparable transcriptional changes in ES. **A** Heatmap illustrates the gene expression profiles of ES cells treated with iP300w (1 µM) for 4 hours, with hierarchical clustering indicating significant concordance among different ES samples. **B** Venn diagram shows the overlap of DEGs in ES cells post P300/CBP inhibition, with a predominant downregulation of gene expression. **C** The Venn diagram of DEGs in three ES cell lines following EWS::FLI1 knockdown reveals both common and unique DEGs across the cell lines [23]. **D** The comparative Venn diagram illustrates the overlap between DEGs in iP300w-treated cells and EWS::FLI1 knockdown cells. **E** The UpsetR comparative analysis shows the overlap of DEGs between ES cell lines and non-ES cell lines treated with iP300w. **F** GSEA enrichment analyses revealed significant enrichment (*p*=8.54e-52) of EWS-FLI1 related pathways (Reactome Riggi) in iP300w-treated ES cell lines compared to other cancer cell lines. The EWS-FLI1 related genes in the “Reactome Riggi” were identified through the overexpression of EWS-FLI1 in human mesenchymal stem cells. **G** Volcano plot showing the distribution of DEGs influenced by the treatment, with EWS::FLI1 binding sites within 50KB of the promoter region, Canonical GGAA microsatellite EWS-ETS activation sites were identified through ChIP-Seq and selected based on their presence in at least 15 out of 18 ES cell lines [23]. **H** GSEA enrichment analyses reveal significant enrichment (*p*=2.18e-4) of cell cycle checkpoints in iP300w-treated ES cell lines (EWS) compared to other cancer cell lines (Other). **I** KEGG pathway enrichment analyses on DEG in iP300w treated ES cells (4 hours). **J** KEGG pathway analyses were conducted on DEGs following EWS::FLI1 knockdown in ES cell lines. Note that iP300w and EWS::FLI1 knockdown both affect the cell cycle and induce cell senescence

Global transcriptional analysis was employed in iP300w-treated ES cells to identify molecular mechanisms underlying EWS:FLI1/P300/CBP-driven malignancy. An advantage of chemical inhibition over siRNA knockdown is its rapid effect. To capture immediate and highly sensitive transcriptional responses while avoiding secondary targets, RNA-Seq was performed at 4 h post-treatment. Comparison revealed distinct clustering based on differentially expressed genes (DEGs), effectively segregating treated cells from controls (Fig. 3A). Principal Component Analysis (PCA) revealed a clear trend: while different ES cell lines clustered together in the control set, they were distinctly separated in the iP300w-treated groups, highlighting the substantial impact of iP300w treatment on cell transcriptomes (Supplementary Fig. 3A, B). Differential gene expression (DGE) analyses revealed a pronounced predominance of downregulated genes over upregulated ones, in a ratio of 4:1, indicating widespread transcriptional repression induced by the treatment (Fig. 3B). This trend remained consistent across all four ES cell lines, demonstrating a notable overlap, especially among the downregulated genes.

To assess the dependency of EWS::FLI1 transcriptional activity on P300/CBP, we compared transcriptional alterations following EWS::FLI1 knockdown (48 h) and P300/CBP inhibition (4 h). Transcriptional profiles of A673, TC71, and SKES1 after EWS::FLI1 knockdown were accessed through the Gene Expression Omnibus (GEO) data repository [23]. Although the knockdown approach requires a much longer timeframe, meaning much greater involvement of indirect targets, Venn diagram analysis indicated a substantial number of common genes affected by EWS::FLI1 knockdown across different ES cell lines, similar to what was observed previously with P300/CBP inhibition (Fig. 3B and C). Subsequent comparison between the two approaches revealed significant overlaps of approximately one-fourth of the differentially expressed genes (DEGs), indicating a shared transcriptional response to both EWS-FLI1 knockdown and iP300w treatment (Fig. 3D).

To underscore the significance of P300/CBP in EWS::FLI1 activity, we utilized UpsetR for a comparative analysis of RNA-Seq data between ES cell lines and non-ES cell lines treated with iP300w (Fig. 3E). In the non-ES cell line pool, we included diverse cancer cell lines such as breast carcinoma (T-47D), rhabdomyosarcoma (A204), synovial sarcoma (Fuji), and osteosarcoma (HOS, U2-OS). The comparison of DEGs revealed that ES cells exhibited heightened sensitivity to iP300w as compared to other cancer cell lines. For instance, 330 DEGs were uniquely affected in ES cells compared to 124 affected genes in other lines (Fig. 3E). Furthermore, Gene Set Enrichment Analysis (GSEA) demonstrated a robust transcriptional correlation between iP300w-treated ES cells and mesenchymal stem cells (MSC) overexpressing EWS::FLI1 (*p* = 8.54e-52; Reactome EWS::FLI1) (Fig. 3F). Notably, 74 of DEGs influenced by the treatment contained EWS::FLI1 binding sites within 50 KB of the promoter region (Fig. 3G). Additionally, GSEA revealed a more pronounced impact on the cell cycle in iP300w-treated ES cell lines compared to other cancer cell lines (*p* = 2.18e-4; Reactome: cell cycle; Fig. 3H). This is further supported by the ATP assay results, which showed that iP300w did not significantly affect the viability of non-ES cell lines (Supplementary Fig. 1A, 3C).

Finally, KEGG pathway analysis revealed modulation of pathways associated with cancer, stem cell pluripotency, and cellular signaling in iP300w-treated cells. Particularly notable changes were observed in the Hippo, Wnt, and Hedgehog pathways, indicating the widespread effects of P300/CBP inhibition on regulatory networks crucial for tumorigenesis and cell differentiation (Fig. 3I). Conversely, in EWS::FLI1 knockdown cells, DNA replication and cell cycle-related pathways were predominantly affected (Fig. 3J). Among the common most affected pathways between iP300w treatment and EWS::FLI1 knockdown were “Transcriptional misregulation in cancer” and “Cellular senescence”. Collectively, these findings highlight that at the transcriptional level, inhibition of P300/CBP with iP300w can yield effects comparable to EWS::FLI1 knockdown in ES cells.

### Differential survival outcomes in ES patients linked to P300/CBP related genes

**Fig. 4:**
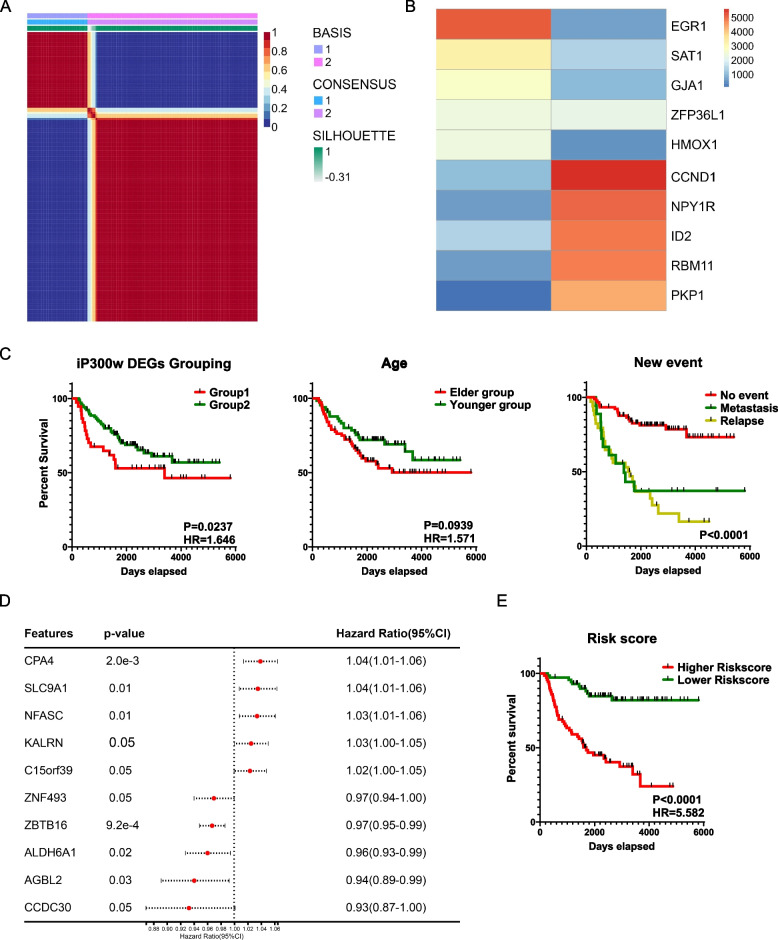
P300/CBP-related genes are linked to poor prognosis in patients with ES. **A** NMF clustering consensus map categorizing 142 ES patients into two distinct groups based on the expression patterns of iP300w-induced DEGs. **B** Heatmap illustrates the expression profiles of the top five genes from each group identified by NMF clustering. **C** Kaplan-Meier survival curves reveal significant differences in survival outcomes between the two patient groups identified through NMF clustering. Additional analyses include survival stratification by median age, as well as metastatic and relapse states. **D** Cox regression analysis presenting the top five and bottom five hazard ratio genes significantly associated with overall survival among the 74 identified genes. **E** The Lasso Cox regression model correlates gene expression with overall survival, assigning risk scores based on gene expression levels. Higher risk scores are identified as significant risk factors

Expanding upon our gene expression findings, we explored the potential clinical relevance of EWS::FLI1/P300/CBP axis-related genes. We correlated the DEGs from iP300w-treated ES cell lines with the overall gene expression profiles of biopsy samples from 142 Ewing sarcoma patients archived in the International Cancer Genome Consortium (ICGC) database and GSE63157 [24]. Employing Non-negative Matrix Factorization (NMF), we categorized these patients into two distinct groups based on the expression patterns of the iP300w affected DEGs. This stratification is visually represented in an NMF clustering consensus map (Fig. 4A), while a heatmap (Fig. 4B) further elaborates on the expression profiles of the top five genes from each group, highlighting the potential impact of these DEGs on patient outcomes.

To explore the clinical implications of our findings, we conducted survival analysis of the two distinct patient groups identified via NMF clustering. There were significant differences (*p* = 0.0237) in survival outcomes among these groups (Fig. 4C), underscoring the potential prognostic relevance of P300/CBP related genes in ES. We stratified the patient samples by median age; however, this categorization did not reveal a significant impact on survival (*p* = 0.0939). Additionally, we assessed the role of metastatic and relapse state in patient prognosis. The analysis revealed that event-fee patients had significantly better survival compared to those with metastasis (Hazard Ratio = 0.4861, *p* = 0.0014) and those with relapse (Hazard Ratio = 0.4608, *p* = 0.0124). However, there was no significant difference in survival between the groups with metastasis and relapse (*p* = 0.913) (Fig. 4C). The overall survival analysis across the three groups was highly significant (*p* < 0.001).

Given the classification of samples into two categories through NMF, a direct understanding of the relationship between DEGs and patient overall survival was not immediately clear. To bridge this gap, we employed Cox regression analysis to examine the link between DEG expression and overall survival, identifying 74 genes significantly associated with patient survival (Wald *p* < 0.05), with the top 5 and bottom 5 hazard ratio genes presented for clarity (Fig. 4D). Recognizing patient survival is unlikely to be regulated by a single gene, we conducted a Lasso Cox analysis of these genes to construct a model correlating gene expression with overall survival (Supplementary Fig. 4). The model assigned scores based on the expression of different genes, and higher scores were identified as a significant risk factor, evidenced by a Hazard ratio of 5.582 and *p* < 0.0001, indicating a strong association with patient survival, comparable to the impact of metastasis or relapse (Fig. 4E).

Our clinical analysis, based on survival data, illuminates the pivotal prognostic role of P300/CBP related genes in ES, revealing a robust correlation between patient stratification according to distinct gene expression patterns and diverse overall survival outcomes. These findings underscore the compelling prognostic relevance of targeting P300/CBP in ES.

### P300/CBP inhibition triggers senescence in ES

**Fig. 5:**
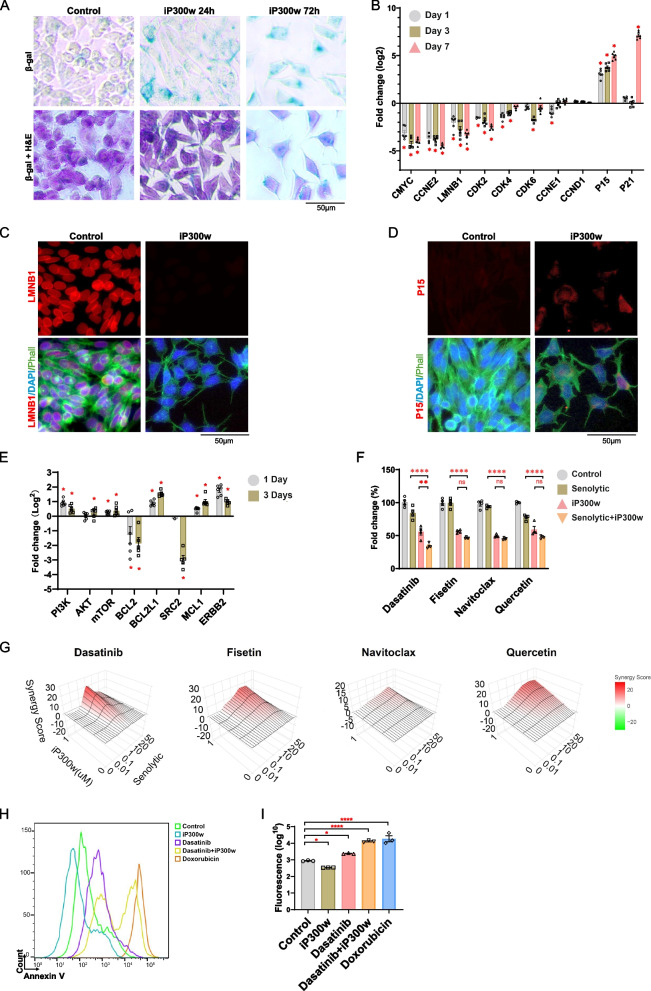
P300/CBP Inhibition triggers senescence in ES cancer cell lines. **A** β-Gal and H&E staining in SKES1 cells at 24 and 72 hours of treatments with iP300w. **B** Gene expression analysis shows changes in proliferation and senescence markers in SKES1 cells following 1, 3, and 7 days of treatment with 1 μM iP300w. Data were normalized to B2M and are presented as fold change compared to the control group. The data represent mean ± SEM; *p*< 0.05, by one-way ANOVA (*n*=6). **C** Immunostaining shows a complete absence of LMNB1 (red) expression in iP300w-treated cells after 72 hours. Phalloidin (green) highlights cell morphology, and DAPI (blue) stains the nuclei. Scale bar: 50 µm. **D** Immunostaining for P15 in cells treated with iP300w for 72 hours. **E** RT-qPCR analysis of senolytics target gene expression following 24 and 72 hours of iP300w (1µM) treatment. Data were normalized to B2M and are presented as fold change compared to the control group. The data represent mean ± SEM; *p<0.05, by one-way ANOVA (*n*=6). **F** ATP assays show cell viability of SKES1 with different senolytics (1 μM) used alone or combined with iP300w (1 μM) following 72 hours of treatment. The data represent mean ± SEM; **p<0.01, ****p<0.0001 by one-way ANOVA. Results are presented as fold change compared to the control group (*n*=4). **G** Synergy scores between iP300w and various senolytics (Dasatinib, Fisetin, Navitoclax, and Quercetin) were calculated using the Highest Single Agent (HSA) model via SynergyFinder Plus. The 3D plots display synergy scores across different concentrations of senolytics (X-axis: 0 μM, 0.01 μM, 0.1 μM, 1 μM, 10 μM, 50 μM) combined with iP300w (Y-axis: 0 μM, 1 μM). The Z-axis represents the synergy score, with positive values indicating synergy and negative values indicating antagonism. The strongest synergistic effects were observed with Dasatinib, while other senolytics demonstrated varying levels of synergy or antagonism. **H** FACS analysis of Annexin V staining (histogram) in SKES1 cells following 72 hours of treatment with iP300w (1 μM), Dasatinib (1 μM), Dasatinib + iP300w, and Doxorubicin (1 μM). **I** Quantification of Annexin V staining. Data are presented as log10 fluorescence intensity. The data represent mean ± SEM; *p<0.05, ****p<0.0001 by one-way ANOVA (*n*=3)

Our findings, indicating iP300w treatment triggers rapid cell cycle arrest and morphological changes characteristic of senescent cells, coupled with KEGG pathway enrichment in Cellular Senescence, motivated us to directly evaluate the relationship between P300/CBP and senescence in ES. We performed β-galactosidase (βGal) staining, a widely recognized marker for cellular senescence, to assess senescence in ES cell lines. A notable increase in β-Gal-positive cells was detected in SKES1 and TC71 24 h after iP300w treatment, even in the absence of evident morphological changes at this stage (Fig. 5A; Supplementary Fig. 5A). Both cell lines exhibited a pronounced increase in β-Gal-positive staining 72 h after treatment. Concurrent Hematoxylin and Eosin (H&E) staining in SKES1 cells revealed significant morphological changes characteristic of senescence, such as enlarged and flattened cell structures, irregular shapes, and increased granularity (Fig. 5A; Supplementary Fig. 5A). To further validate our findings, we confirmed senescence induction by iP300w through β-Gal staining in additional EWS::FLI1-driven Ewing sarcoma cell lines, including CHLA25, A4573, and CHLA9 (Supplementary Fig. 5B). We then expanded our analysis to track early and late senescence-associated gene expression over a seven-day period in these treated cell lines. Although cell cycle gene alterations highlighted a significant impact on proliferation, it was surprising to observe that the early senescence markers P16 and P21 in most cases were not upregulated (Fig. 5B; Supplementary Fig. 5C). Upon broadening our panel of senescence-related markers, we noted a considerable decrease in LMNB1 expression in all ES cell lines or xenograft tumors treated with iP300w (Figs. 2I and 5B). This effect was further confirmed at the protein level by immunofluorescence (Fig. 5C and D; Supplementary Fig. 5D). Interestingly, we observed that only the ES cell lines harboring functional P53 mutations (SKES1, TC71, CHLA25) responded with P15 induction upon iP300w treatment, whereas the cell lines with intact P53 (A4573 and CHLA9) did not show this response (Fig. 5B; Supplementary Fig. 5C). Remarkably, alterations in the expression of P15 was observed as early as 4 h into the treatment (Supplementary Fig. 7A).

To determine whether P300/CBP inhibition also induces senescence in ES cell lines driven by EWS::ERG translocation, we extended our analysis to the CADO-ES1 and COG-E-352 cell lines. Initial results showed that iP300w treatment reduced cell viability in EWS::ERG cells, though this effect was less pronounced compared to EWS::FLI1 cell lines at 48 h, as measured by the ATP assay (Supplementary Fig. 6A). Notably, after extending the treatment to 72 h, we observed a complete loss of Ki67 staining, indicating a profound effect on cell proliferation (Supplementary Fig. 6B). Similarly to the EWS::FLI1 cell lines, both CADO-ES1 and COGE352 lines underwent senescence following iP300w treatment. This was evidenced by changes in cell morphology, expression of senescence-related genes, and β-Gal positivity (Supplementary Fig. 6C). Downregulation of LMNB1 was consistently observed as a hallmark of the senescence process (Supplementary Fig. 6C, E, F, H).

We then reasoned that if senescence is indeed one of the initial responses to P300/CBP inhibition, senolytics might facilitate the clearance of iP300w-treated ES cells. Senolytics comprise a group of drugs that target various senescence-related pathways [25]. To identify the most appropriate senolytic, we assessed the commonly affected PI3K and BCL-2 pathways by RT-qPCR. Gene expression analyses revealed a significant upregulation of PI3K, mTOR, MCL-1, and ERBB2, and a reduction of BCL-2 and SRC2 (Fig. 5E). RNA-Seq data suggested signs of these changes were apparent as early as 4 h following P300/CBP inhibition (Supplementary Fig. 7A). Hence, for our testing, we opted for Dasatinib and Fisetin, established senolytics targeting the PI3K pathways, while Navitoclax and Quercetin, known BCL-2 targets, served as negative controls [25–27]. We proceeded to assess the potential synergistic effects of these senolytics when combined with iP300w, testing each senolytic across a concentration range from 0 to 50 μM. Dasatinib displayed an IC50 between 1–10 μM, while Fisetin and Navitoclax fell within the 20–50 μM range (Supplementary Fig. 7B, C). Quercetin showed no effectiveness even at the highest tested concentrations. Combining iP300w with these senolytics significantly reduced cell viability, as evidenced by the percentage fold change compared to the control group in the ATP assay (Supplementary Fig. 7B, C). Particularly noteworthy was the marked improvement observed with the combination of Dasatinib with iP300w that resulted in a 70% inhibition of cell viability at 1 μM concentrations for both agents (Fig. 5F; Supplementary Fig. 7D). This underscores a potent synergistic effect when iP300w is used alongside selected senolytics. To quantitatively assess the synergistic effects of combining iP300w with senolytics, we utilized SynergyFinder Plus. Our analysis revealed Dasatinib at 1 μM, when combined with 1 μM of iP300w, achieved the highest synergy score (Fig. 5G; Supplementary Fig. 7E). After confirming that the combination of Dasatinib with iP300w exhibited the strongest synergistic effect, we proceeded to evaluate the ability of this combined treatment to induce apoptosis. While Dasatinib alone caused only minimal apoptosis in SKES1 cells, the addition of iP300w significantly enhanced this effect, achieving levels comparable to those induced by Doxorubicin (Fig. 5H, I; Supplementary Fig. 7F, G). Bringing our findings together, we concluded that ES undergoes senescence as an early response to P300/CBP inhibition, characterized by alterations in LMNB1 and PI3K expression.

### Regulation of LMNB1 by the EWS::FLI1

The noncanonical senescence pathway induced by iP300w in ES prompted further investigation into the interplay between LMNB1/P15 and EWS::FLI1/P300/CBP transcriptional axis. To explore this, we leveraged previously published high-resolution ChIP-Seq data obtained from studies examining the epigenetic effects of gain and loss of EWS::FLI1 function in ES and MCS [8, 16].

**Fig. 6:**
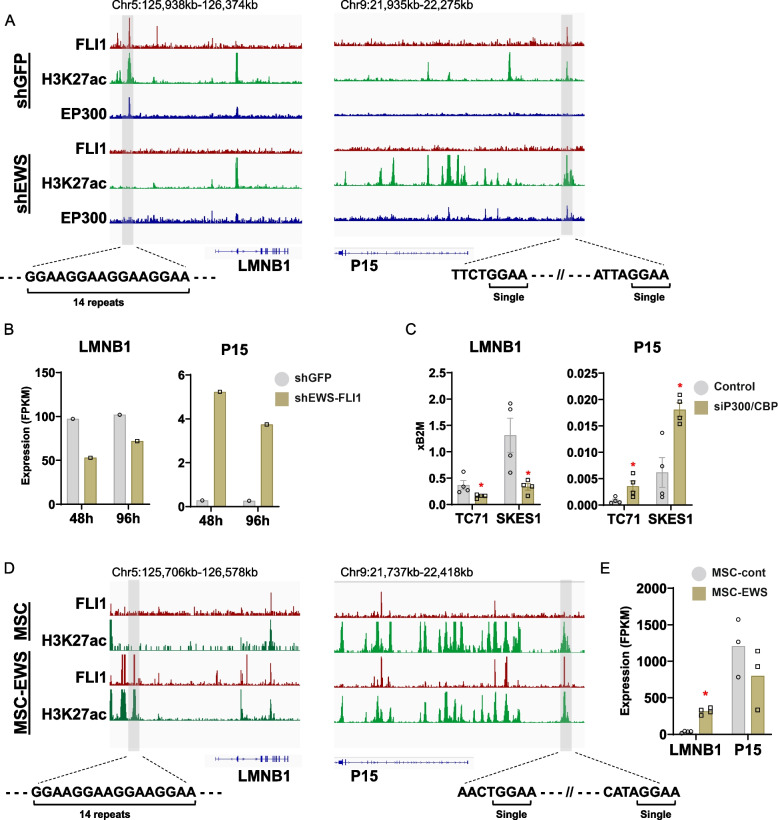
EWS::FLI1/ regulates LMNB1 and P15 and cellular senescence in ES. **A** ChIP-Seq analysis reveals that EWS::FLI1 binds to regulatory elements of LMNB1 and P15 in the SKNMC cell line [8]. Specifically, EWS::FLI1 targets the repetitive GGAA motif in the regulatory elements of LMNB1 and a single motif in P15. EWS::FLI1 binding sites in LMNB1 are associated with P300 and acetylation of H3K27. **B** Transcriptional levels (RNA-Seq) of LMNB1 and P15 following EWS::FLI1 knockdown (96 hours) reveals reduction of LMNB1 and induction of P15. **C** RT-qPCR for LMNB1 and P15 expression 48 hours post P300/CBP knockdown. Data were normalized to B2M and are presented as fold change compared to the control group. The data represent mean ± SEM; *p<*0.05 by t-test (*n*=4). **D** ChIP-Seq data shows EWS::FLI1 binding in LMNB1 and P15 regulatory elements in MSC overexpressing EWS::FLI1 [16]. **E** LMNB1 and P15 expression (RNA-Seq) in MSC overexpressing EWS::FLI1 and GFP control cells. Data are presented as mean expression levels. The data represent mean ± SEM; *p<*0.05 by t-test (*n*=4)

Analysis of ChIP-Seq data revealed EWS::FLI1 binding in the regulatory elements of both LMNB1 and P15. The binding region in LMNB1 comprises consecutive GGAA repeats (more than 14), which are associated with EWS::FLI1 transcriptional activation, while the binding site in P15 features a single GGAA motif that facilitates oncogene transcriptional repression (Fig. 6A). Notably, the EWS:FLI1 peak in LMNB1 is also associated with acetylated H3K27 and P300, indicating active chromatin. Upon EWS::FLI1 knockdown, the EWS:FLI1 binding is lost as expected, along with simultaneous loss of P300 binding and reduction of H3K27 acetylation (Fig. 6A). The epigenetic alterations in the LMNB1 enhancer are reflected in the transcriptional levels, as the gene is downregulated by EWS:FLI1 knockdown (Fig. 6B). Contrary to LMNB1, EWS::FLI1 binding of P15 was not accompanied by P300 and maintained low H3K27 acetylation. Upon EWS::FLI1 knockdown, the repression of the locus was lost, as indicated by increased acetylation and gene induction (Fig. 6A and B). To confirm EWS::FLI1 transcriptionally regulates LMNB1 through P300/CBP, we reassessed our data from experiments in which we knocked down both proteins (Fig. 1). Indeed, the transcription of LMNB1 upon P300/CBP knockdown mirrored those observed with EWS::FLI1 knockdown (Fig. 6C).

Overexpression of EWS::FLI1 in non-permissive cells leads to induction of cell cycle arrest and cell death [14]. Therefore, we hypothesized the opposite mechanism might apply when EWS::FLI1 is expressed in a permissive environment. To investigate this, we retrieved ChIP-Seq data in which EWS::FLI1 was overexpressed in MSC to promote malignant transformation [16, 28]. As predicted, EWS::FLI1 overexpression resulted in EWS::FLI1 binding to the repetitive GGAA motif, facilitating H3K27 acetylation and LMNB1 expression (Fig. 6D and E). Additionally, EWS-FLI1 binds to the repressive single GGAA motif, leading to reduced H3K27 acetylation at the P15 locus (Fig. 6D). In summary, our findings demonstrate the EWS::FLI1/P300/CBP axis directly regulates the expression of key senescence-related genes LMNB1, promoting cell viability and preventing senescence in Ewing sarcoma and permissive cells.

To further investigate whether EWS::FLI1 and EWS::ERG-mediated senescence evasion mechanisms operate independently of the canonical TP53 and P16/P21 pathways, we examined the binding sites of these oncoproteins at their regulatory regions in SKNMC, SKES1, and TC32 cells. Our analysis revealed that both EWS::FLI1 and EWS::ERG consistently bind only to LMNB1, with no direct interactions observed with TP53, P16, or P21. This suggests that any transcriptional activity of P16, P21, and TP53 might be independent of these oncoproteins (Supplementary Fig. 8A-E). Additionally, the previously observed binding of EWS::FLI1 to the repressive motif of P15 in SKNMC was also identified in SKES1 cells, both of which have nonfunctional P53. Notably, EWS::FLI1 binding to P15 is not associated with P300 peaks, indicating a P300/CBP-independent regulatory mechanism (Supplementary Fig. 8A). In summary, we demonstrate that both EWS::FLI1 and EWS::ERG cooperate with P300 to maintain LMNB1 expression and evade senescence-related processes in Ewing sarcoma.

### LMNB1 and P15 suppress apoptosis and sustain iP300w induced senescence

**Fig. 7:**
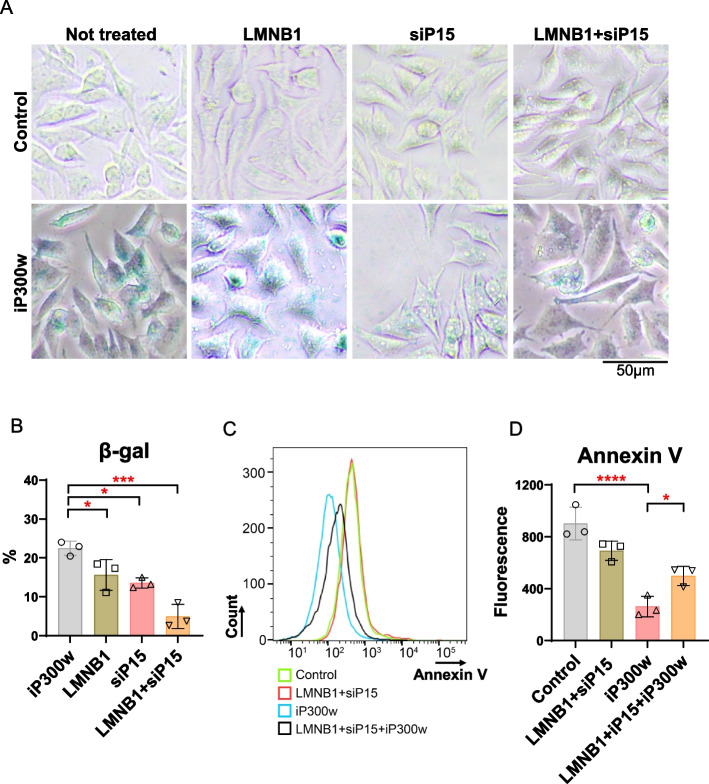
The restoration of LMNB1 and P15 expression in iP300w-treated cells delays senescence and induces apoptosis. **A** β-Gal staining was performed on SKES1 cells treated with iP300w (1 µM) for 48 hours, as well as on control untreated cells. LMNB1 refers to SKES1 cells that constitutively overexpress LMNB1 from a viral construct, while siP15 denotes SKES1 cells with P15 knocked down. LMNB1+siP15 refers to the condition where LMNB1 is expressed and P15 is knocked down for 48 hours. **B** Quantification of β-Gal positive cells presented in “A”. The data represent mean ± SEM; *p<0.05, ***p<0.001 by one-way ANOVA (*n*=3). **C** Annexin V staining (histogram) of SKES1 cells in the condition described above. **D** Quantification of intensity of Annexin V staining. The data represent mean ± SEM; *p<0.05, ****p<0.0001 by one-way ANOVA (*n*=3)

To assess the functional relevance of iP300w induced senescence mediated by LMNB1/P15 in ES cells with non-functional P53, we conducted gene rescue experiments. Our assumption was that simultaneous overexpression of LMNB1 and knockdown of P15 would delay the senescence. To achieve constitutive LMNB1 overexpression, SKES1 cells were transduced with a lentiviral vector carrying LMNB1, while P15 was knocked down using siRNA (Supplementary Fig. 9A). β-Gal staining revealed a significant induction in 21% of the cells by 24 h of iP300w treatment, a process notably reduced to 4% upon overexpression of LMNB1 and knockdown of P15 (Fig. 7A, B).

Senescent cells evade apoptosis through a variety of mechanisms, such as enhancing anti-apoptotic pathways, modifying pro-survival signaling, and regulating apoptotic regulators, enabling them to persist in a senescent state [29]. We conducted Annexin V staining to evaluate the apoptotic status in LMNB1/P15 rescued cells. As anticipated, SKES1 cells treated with iP300w exhibited a significant reduction in fluorescence signal and a marked decrease in Annexin V-positive cells (Fig. 7C, D; Supplementary Fig. 9B, C). However, following LMNB1 overexpression and P15 RNA interference, both the fluorescence intensity and the proportion of Annexin V-positive cells were partially reversed (Fig. 7C, D; Supplementary Fig. 9B, C). These functional experiments provide direct evidence that LMNB1 and P15 play a critical role in iP300w-induced senescence.

## Discussion

**Fig. 8:**
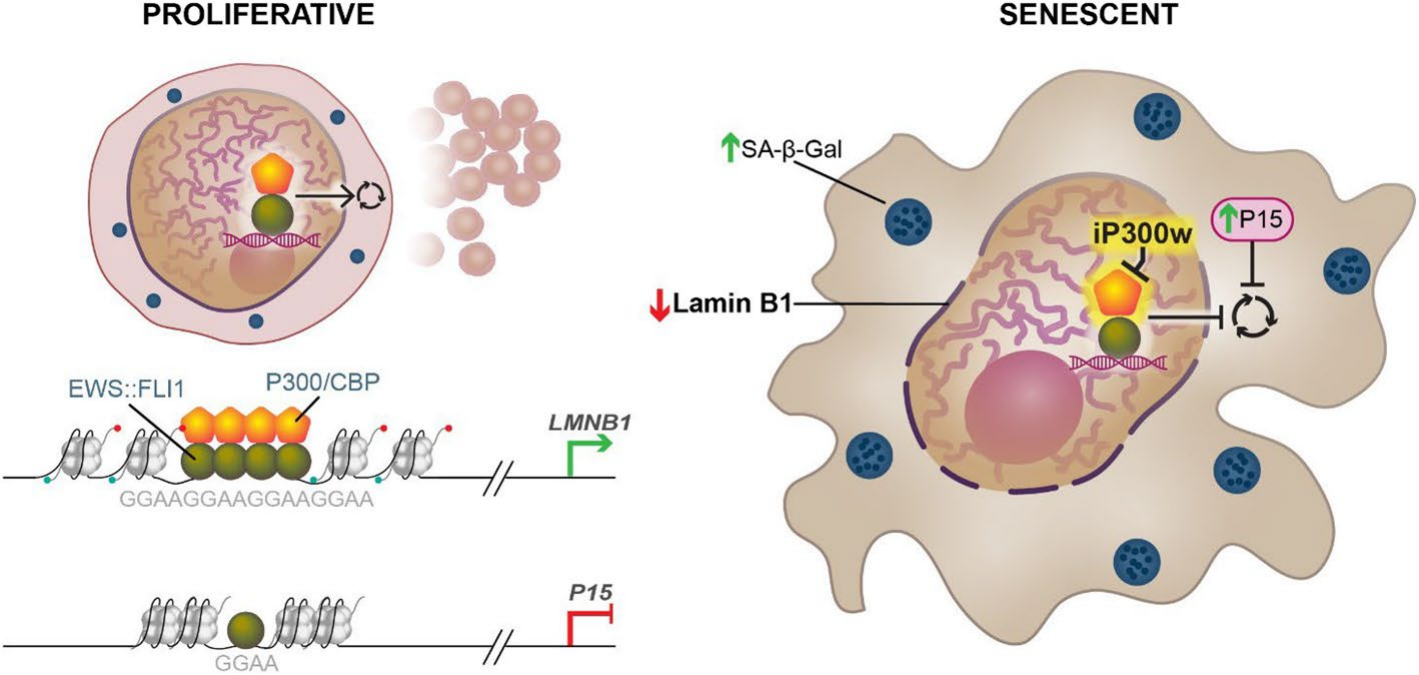
The proposed mechanism by which EWS::FLI1 regulates LMNB1 in ES cancer cell lines. In proliferative ES cells, the EWS::FLI1/P300 complex induces LMNB1 by binding to its enhancer motif containing 14 GGAA repeats. Inhibition of P300/CBP by iP300w treatment suppresses EWS::FLI1 transcriptional activity, leading to reduced LMNB1 transcription. Consequently, SA-β-Ga and other early senescence markers are induced. In a subset of ES cancer cell lines with non-functional P53, EWS::FLI1 binds to a single GGAA motif in the regulatory elements of P15, independent of P300, and represses its transcription. Following treatment with iP300w and subsequent LMNB1 suppression, P15 is rapidly induced, contributing to the induction of senescence in ES cells

This study unveils the critical mechanism of the EWS::FLI1/P300/CBP axis in maintaining ES viability and exposes its vulnerability for pharmacological targeting by the P300/CBP inhibitor, iP300w. Additionally, we elucidate a novel mechanism by which EWS::FLI1 prevents ES or permissive MSC from undergoing senescence by controlling the expression of LMNB1. EWS::FLI1, together with P300/CBP induce LMNB1 transcription by binding to multiple GGAA repeats in its regulatory elements. P300/CBP inhibition rapidly inactivated EWS::FLI1 transcriptional activity, leading to the downregulation of LMNB1, consequently inducing cell senescence (Fig. 8). Finally, we demonstrate that PI3K-targeting senolytics effectively eliminate iP300w-induced senescent ES cells, suggesting a novel, combined therapeutic approach.

While prior studies have shown EWS::FLI1 can recruit P300 to differentially regulate super enhancers [8], the role of P300/CBP in ES viability remains undefined [15]. To explore this, we initially knocked down P300/CBP using siRNA and demonstrated that the oncogenic activity of EWS::FLI1 largely depends on these acetyltransferases. Prompted by our initial results and recognizing the translation limitations of the siRNA approach [30], we investigated pharmacological targeting of P300/CBP, capitalizing on the new generation of more specific spirocyclic P300/CBP inhibitors, including iP300w [19, 31]. We initially described iP300w as an effective inactivator of DUX4, a pioneer transcriptional factor that recruits P300/CBP through its C-terminal domain and plays a key role in early embryonic development and facioscapulohumeral muscular dystrophy (FSHD) [31, 32]. The translocation of the DUX4 C-terminus transforms CIC from a transcriptional repressor to an activator, thereby driving the development of CIC::DUX4 sarcoma. Notably, iP300w displayed superior efficacy against CIC::DUX4 sarcoma compared to A-485 [18], another P300/CBP inhibitor from the same spirocyclic chemical series found to be effective against certain hematological malignancies, androgen receptor-positive prostate cancer, and NUT midline carcinoma [19, 33]. Likewise, iP300w demonstrated high efficacy against ES, evidenced by rapid induction of cell arrest and inactivation of EWS::FLI1 transcriptional activity, comparable to the effects seen with knockdown of EWS::FLI1 [23, 34]. The observed effect was specific to ES cells, with minimal reduction in cell viability noted in human myoblasts and osteosarcoma cell lines at the tested concentrations. Furthermore, the extent of global transcriptional alterations in ES cell lines treated with iP300w surpassed that observed in synovial sarcoma, rhabdomyosarcoma, or osteosarcomas. Our findings underscore the pivotal role of P300/CBP in ES malignancy, as evidenced by a robust correlation between patient stratification based on specific EWS::FLI1/P300/CBP-related gene expression patterns and overall survival rates. Notably, this correlation demonstrates greater predictive power for survival outcomes than traditional clinical features [35]. Similarly, elevated levels of P300 have been identified as indicators of high malignancy and poor prognostic survival factors in liver, nasopharyngeal, and prostatic cancers [36–38].

Despite extensive research over the decades, treatment options for Ewing sarcoma) remain limited. FDA-approved drugs such as vincristine, doxorubicin, and cyclophosphamide show some effectiveness but lack specificity for the EWS::FLI1-driven malignancy [39–41]. These are drugs that target DNA replication and cell proliferation, leading to unintended effects on healthy cells. Ifosfamide, another commonly used chemotherapeutic agent for ES, is an alkylating agent that cross-links DNA strands [42]. It is associated with significant side effects, including renal toxicity, cardiotoxicity, and neurotoxicity. Etoposide and topotecan, both topoisomerase inhibitors, are used in the treatment of various cancers, including ES, but their use can also lead to serious side effects such as secondary leukemias and myelosuppression [43, 44]. Other drugs that have shown some effectiveness for ES, when used in combination with other treatments, include irinotecan, actinomycin D, regorafenib, clofarabine, cladribine, and trabectedin [40, 45–48]. Recently, efforts to target the EWSR1::FLI1 fusion or its downstream pathways have been largely unsuccessful. For instance, TK-216, a pharmacological agent designed to disrupt the interaction between EWSR1::FLI1 and RNA helicase A, demonstrated responses in 3 of 85 patients in a phase I/II clinical trial, with an overall disease control rate of 25% and a median duration of response of 25.5 months [49]. Similarly, trabectedin, a DNA minor groove-binding agent, has demonstrated minimal clinical activity as a single agent despite its effects on EWSR1::FLI1 transcriptional targets [50]. Consequently, there is a critical need for therapies capable of specifically targeting EWS::FLI1 activity. The current study demonstrates that inhibiting P300/CBP can inactivate the EWS::FLI1 oncogenic axis, making it a rationally targeted pharmacological approach for treating ES.

Pathway analyses of differentially expressed genes alerted by iP300w revealed activation of the senescence pathway early in the treatment. The senescence phenotype was further evidenced by altered cell morphology, reduced cell proliferation, β-Gal positive staining, and changes in gene expression related to senescence. Senescence induced by P300/CBP inhibition, whether via small molecules or knockdown, has been previously observed in various cancers, such as melanoma, non-small cell lung cancer, and breast cancer [51–53]. This effect is primarily attributed to global changes of chromatin acetylation, with subsequent impact on the regulatory elements of senescence-related genes, among others [54]. Conversely, recent reports indicate P300 inhibition prevents age-related cellular senescence, primarily driven by P300, inducing a dynamic, hyper-acetylated chromatin state and promoting the formation of active enhancer elements in the non-coding genome [55]. The rapid induction of senescence in ES by iP300w suggests a more specific interplay. This prompted further mechanistic exploration, uncovering a novel mechanism by which EWS::FLI1 directly and concurrently regulates the expression of LMNB1. Surprisingly, the classical senescence pathways typically mediated by P16 or P53/P21 were not affected by iP300w in ES [56, 57].

EWS::FLI1 exhibits distinct binding preferences depending on the arrangement of GGAA motifs within regulatory regions. When encountering repetitive GGAA motifs, EWS:FLI1 tends to promote gene activation. In contrast, when encountering single GGAA motifs, it tends to facilitate gene repression [8, 16]. We found a novel mechanism by which EWS::FLI1 directly abrogates senescence by activating LMNB1 through binding to repetitive GGAA motifs in its regulatory elements, thereby recruiting P300 and inducing H3K27 acetylation. Conversely, in the subset of cell lines harboring P53 mutation, it inhibits P15 independently of P300 through sites containing a single GGAA motif. Previous studies have suggested various mechanisms by which EWS::FLI1 indirectly evades senescence, such as destabilizing P27 via Skp2 and suppressing P53 and P21 through the NOTCH signaling pathway [58–60]. It is well-documented that overexpression of EWS::FLI1 induces growth arrest and apoptosis in the majority of cell types, except for MSCs and neuroectodermal cells [12]. However, deletion of p53, p19, and p16 in mouse embryonic fibroblasts attenuates apoptosis and promotes EWS::FLI1-driven transformation [14, 61]. Intriguingly, we also discovered that EWS::FLI1 directly controls LMNB1 and P15 in permissive MSC, potentially averting cell arrest, preventing senescence and fostering malignant transformation. Loss of LMNB1 expression has been a well-documented trigger of senescence, a cellular state often characterized by the accumulation of P15 protein [62, 63]. Additionally, LMNB1 affects both proliferation and senescence, potentially through a ROS signaling pathway, leading to subsequent changes in chromatin, such as the enrichment of H3K4me3 and H3K27me3 [55, 64]. We further confirmed the functional relevance of LMNB1 and P15 in iP300w-mediated ES senescence through gene rescue experiments. By overexpressing LMNB1 and knocking down P15 in treated cells, we observed that reconstitution of LMNB1 and P15 expression delayed iP300w-induced senescence and promoted apoptosis. This was evidenced by a reduction in βGgal staining and an increase in Annexin V positive cells. Resistance of senescent cells to apoptosis occurs primarily through alterations in P53 [65]. They maintain low levels of P53 due to decreased stabilization and exhibit senescence-specific post-translational modifications of P53. These adaptations prevent the activation of P53-mediated apoptotic pathways, even in the presence of typical apoptotic stimuli like actinomycin D, low-dose cisplatin, and staurosporine [65].

Building on the success of inducing senescence in breast and lung cancer xenografts, followed by elimination with senolytics [66], we explored a comparable strategy to target iP300w-treated ES cells. Our study revealed that P300/CBP inhibition activates the PI3K and suppresses BCL-2 pathways. To exploit this vulnerability, we selected Dasatinib, a senolytic targeting the PI3K pathway, for the targeted elimination of senescent cells [25]. Conversely, senolytics targeting the BCL-2 pathway, like Navitoclax [25], were completely ineffective in eliminating the senescent cells, further solidifying the involvement of the PI3K pathway.

Dasatinib, a tyrosine kinase inhibitor, functions by inhibiting SRC or RTKs, consequently affecting the PI3K/AKT pathway [67]. The activation of the PI3K/AKT pathway broadens the target scope of Dasatinib, resulting in a synergistic effect when combined with iP300w. However, the initial decrease in BCL-2 expression in senescent cells might suggest a reduced target scope for BCL-2-targeting senolytics like Fisetin, Navitoclax, and Quercetin [25]. Nonetheless, the subsequent increase in MCL-1 levels can provide a compensatory survival mechanism for these cells. MCL-1, unique among the BCL-2 family, is responsible for conferring resistance to chemotherapeutic agents [26]. The upregulation of MCL-1 could attenuate the effects of BCL-2 inhibition, potentially accounting for the reduced synergy observed with P300/CBP inhibitors [26].

In conclusion, we have identified a novel mechanism by which EWS::FLI1 evades cellular senescence, pinpointing EWS:FLI1/P300/CBP as a vulnerable target in ES (Fig. 8). Furthermore, we have demonstrated the feasibility of pharmacological targeting of P300/CBP in ES. While we utilized the pharmacological compound iP300w in this study, similar effects could potentially be achieved with other recently discovered P300/CBP inhibitors such as A-485, CCS1477, NEO2734, and B026 which have shown effectiveness against various malignancies, with some even undergoing clinical trials [19, 68–70]. In addition, we propose a more potent approach that integrates P300/CBP inhibition with senolytic agents to target newly senescent cells for elimination. By employing this dual approach, we echo successful methodologies used in other cancer contexts and position it as a forward-looking avenue for ES treatment. Our study delineates the critical roles of LMNB1 within this axis and demonstrates the efficacy of combining P300/CBP inhibition with senolytics, illuminating a potentially transformative approach to ES therapy. It underscores the intricate interplay of molecular pathways in ES and highlights the promising therapeutic implications of their modulation.

## Materials and methods

### Cell culture and treatments

All basal media were acquired from HyClone, fetal bovine serum (FBS) was sourced from PeakSerum (Ps-FB3, lot 293Q16), and both Glutamax (Glu) and Penicillin/Streptomycin (P/S) were obtained from GIBCO. The ES cell lines TC71, A673, A4573, and SKES1, a generous gift from Dr. Michael Verneris, University of Minnesota, were cultured in DMEM supplemented with 10% FBS, Glu, and P/S. The ES cell lines CHLA9, CHLA25, and COG-E-352, a gift from the Childhood Cancer Repository, were cultured in DMEM supplemented with 20% Fetal Bovine Serum, 4 mM L-Glutamine, and 1X ITS (5 µg/mL insulin, 5 µg/mL transferrin, 5 ng/mL selenous acid). The ES cell line CADO-ES1, purchased from Cytion (Gaithersburg, US), was cultured in DMEM: Ham's F12 supplemented with 15 mM HEPES. The breast carcinoma (T-47D) cell line was gift from Dr. Reuben Harris, University of Minnesota, rhabdomyosarcoma (A204) cell line was gift from Dr. Dennis Wigle, Mayo Clinic, synovial sarcoma (Fuji), osteosarcoma cell lines U2-OS, 143B, SJSA-1, HOS, and G292, also a gift from Dr. Beau Webber, University of Minnesota, were cultured in DMEM supplemented with 10% FBS, Glu, and P/S. The LHCN-M2 cell line, representing immortalized human myoblasts, was cultured in a proliferation medium composed of F10 supplemented with 20% FBS, 2-mercaptoethanol at a concentration of 1 × (GIBCO), 10 − 9 M dexamethasone (Sigma), 10 ng/mL bFGF (Peprotech), along with Glu/P/S [32]. All cell cultures were maintained at 37 °C in a 5% CO2 atmosphere.

### Cell viability (ATP) assay and synergy calculation

Cell lines were plated in 96-well plates at a density of 1 × 10^5^ cells per well. The following day, iP300w or its stereoisomers were administered as part of the therapeutic regimen. Cell viability was assessed using the CellTiter-Glo® Luminescent Cell Viability Assay kit (Promega), adhering closely to the manufacturer's guidelines. Luminescence measurements, indicative of cell viability, were taken using the POLARstar Optima Microplate Reader (BMG Labtech, Offenburg, Germany).

Senolytic agents Dasatinib (SML2589, Sigma-Aldrich), Quercetin (73932, STEMCELL Technologies), Fisetin (S2298, Selleck Chemicals), and Navitoclax (NC1731582, Fisher Scientific) were diluted with DMSO and medium to concentrations of 0.01 μM, 0.1 μM, 1 μM, 10 μM, 20 μM, and 50 μM. In the control group, DMSO concentration was adjusted to match the highest treatment concentration of 50 μM. Both senolytics and iP300w were applied either alone or in combination for a duration of 72 h, followed by ATP assay analysis as previously described.

Following the completion of the ATP viability assays, data were imported into SynergyFinder Plus for synergy score analysis. Using the Highest Single Agent (HSA) model, we calculated synergy scores across the tested conditions, focusing on the combined effects of iP300w (0 μM, 1 μM) and senolytics (Dasatinib, Quercetin, Fisetin, and Navitoclax) across a range of concentrations (0 μM, 0.01 μM, 0.1 μM, 1 μM, 10 μM, 20 μM, and 50 μM). The analysis was performed using the default settings of the HSA model, which compares the efficacy of combination treatments relative to the most effective single-agent treatment in each condition.

### Antibodies, western blot, and immunofluorescence

For western blot analyses, cell lysates were prepared using RIPA buffer enhanced with a protease inhibitor cocktail (Complete, Roche). Proteins were resolved by electrophoresis on 10% SDS-PAGE gels and subsequently transferred to PVDF membranes. The membranes were incubated with primary antibodies diluted in 5% skim milk in TBST either overnight at 4 °C or for 1 h at room temperature (RT). Following primary antibody incubation, membranes were treated with an appropriate HRP-conjugated secondary antibody for 1 h at RT. After washing with TBST, the protein bands were visualized using Pierce ECL western blotting substrate (Thermo Scientific).

For immunofluorescence assays, cells grown in 96-well plates were fixed with 4% paraformaldehyde (PFA) for 10 min, washed twice with PBS, and then permeabilized with 0.3% Triton X-100 for 30 min. Blocking was performed with 3% BSA for 1 h at room temperature. Primary antibodies were diluted in 3% BSA and incubated overnight at 4 °C. Subsequently, cells were incubated with a suitable fluorophore-conjugated secondary antibody for 60 min at room temperature. Nuclei staining was achieved using DAPI (dilution 1:5000, Sigma).

The antibodies utilized in this study included GAPDH-HRP (1:5000, Proteintech 60004), rabbit anti-Histone H3K18Ac (1:500, Abcam ab1191), rabbit anti-Histone H3K27Ac (1:500, Abcam ab1791, lot: GR3297878-1), rabbit anti-LMNB1 (1:1000, Abcam ab16048), rabbit anti-γH2AX (1:1000, Cell Signaling 9718S), rabbit anti-Ki-67 (dilution 1:250, Cell Signaling 9129T), anti-mouse P300 (1:500, Active Motif 61,401), rabbit anti-CBP (1:1000, Cell Signaling 7389S), secondary Alexa Fluor 555 Goat Anti-Rabbit (1:500, Invitrogen), HRP-conjugated anti-rabbit (1:5000, Jackson Immuno Research 111–035-003, lot: 149,393), and HRP-conjugated anti-mouse (1:2500, Novus NBP1-75,130, lot 58–173-090418).

### In vivo mouse tumor formation and iP300w evaluation

Mouse tumor formation and evaluation of iP300w in vivo were carried out at the University of Minnesota Research Animal Resources facility, adhering to protocol (2209-40422A) approved by IACUC. Mice were grouped by matching sex and age, with random assignments to control or experimental groups. Immunodeficient NSG mice, three months old, were transplanted with 0.56 × 10^7^ SKES1 cells in 100 µL of medium and Matrigel (Corning) mix. iP300w was prepared in DMSO (10 mM), diluted in 100 µL PBS, and administered intraperitoneally twice daily, with the control group receiving the vehicle only. Treatment was initiated one day after transplantation. Tumor size was measured every 3 days, using the formula 0.5 × length × width^2^. Mice were identified by number, and the investigator was blinded to the treatment status during tumor dissection, weighing, and photographing.

### RNA isolation, quantitative real-time RT-PCR, and RNA sequencing

RNA was isolated employing the Zymo RNA extraction kit, and complementary DNA (cDNA) was synthesized from 0.5 µg of total RNA using an oligo-dT primer and the Verso cDNA Synthesis Kit from Thermo Scientific, adhering strictly to the provided protocol. Quantitative PCR (qPCR) analyses utilized Premix Ex Taq (Probe qPCR, Takara) or SYBR Green for detection. Gene expression quantification was anchored to GAPDH or B2M reference genes and computed using the 7500 System Software through the ΔCT method by Applied Biosystems. Primers and probes are listed in Table 1. For RNA sequencing (RNA-Seq), libraries were prepared from 500 ng of total RNA extracted from cells treated with iP300w (1.0 µM) for 4 h. Library preparation and sequencing (2 × 150 bp, 20M paired-end reads) was done at Azenta Life Science, USA.

**Table 1:** Primers and probes

Gene	Forward Primer (F)	Reverse Primer (R)
EWS::FLI1	ATT GCC CCA AGC TCC TCT TC	ATT GCC CCA AGC TCC TCT TC
P300	GGC TGT ATC AGA GCG TAT TGT C	CCT CGA AAT AAG GCA ATT CC
CBP	GTC CAG TTG CCA CCA GCA C	CAT TCG GGA AGG AGA AAT GG
NR0B1	TTT CTT TCC AAA TGC TGG AGT CTG A	GAA TGT ACT TCA CGC ACT GCA G
ID2	GCC CAG CAT CCC CCA GAA	GGT GGT CAG CGG CGT CCT
MEIS1	GCG CAA AGG TAC GAC GAT CT	GGT ACT GAT GCG AGT GCA GA
NKX2.2	GAA CCC CTT CTA CGA CAG CA	GGG TCT CCT TGT CAT TGT CC
CDK2	TAC CAC AGG GTC ACC ACC TC	TCC TCC ACC GAG ACC TTA AA
CCND1	CAT CTA CAC CGA CAA CTC CAT C	GGA AGC GGT CCA GGT AGT TC
LMNB1	GGG AAG TTT ATT CGC TTG AAG A	ATC TCC CAG CCT CCC ATT
P16	CCC AAC GCC CCG AAC T	GCA GAA GAG CTG CTA CGT GAA
CDK4	GTG TAT GGG GCC GTA GGA AC	CAG TCG CCT CAG TAA AGC CA
CCNE2	GGG AAA CAT TTT ATC TTG CAC A	CTG CAA GCA CCA TCA GTG AC
JARID2	TTG CCT CGT TCG TCT TTG GC	TCC CAT CAC TGT CAT CGT ATT TCT
APCDD1	AAT GCC AAG AAC CAC GAC CA	GAA GAT GAA GTG GCG GGT GA
STEAP1	TGG GCA TAT CAA CAG GTC CAA	AAT GCG TGT ATT GTG CCC AG
P21	GTC AGG CTG GTC TGC CTC CG	CGG TCC CGT GGA CAG GAG CAG
BCL2	TCGCCCTGTGGATGACTGA	CAGAGACAGCCAGGAGAAATCA
PI3K	GGTTGTCTGTCAATCGGTGACTGT	GAACTGCAGTGCACCTTTCAAGC
AKT	TTCTGCAGCTATGCGCAATGTG	TGGCCAGCATACCATAGTGAGGTT
mTOR	GCTTGATTTGGTTCCCAGGACAGT	GTGCTGAGTTTGCTGTACCCATGT
SRC1	ATCACCGCAAGAGCTACCATT	TGACGGTGTCCGAGGAGTTG
SRC2	CCTCCCGTGCGTCCGTCT	AGCCGCTCGCCTTTCTT
ERBB2	CCTCTGACGTCCATCATCTC	ATCTTCTGCTGCCGTCGCTT
MCL1	GGACATCAAAAACGAAGACG	GCAGCTTTCTTGGTTTATGG
BCL2L1	GGAGAACGGCGGCTGGGATA	GGCCACAGTCATGCCCGTCA
P15	GAATGCGCGAGGAGAACAA	CATCATCATGACCTGGATCGC
CDK6	TGGAGACCTTCGAGCACC	CACTCCAGGCTCTGGAACTT
BCL11B	GGC GAT GCC AGA ATA GAT GCC G	CCA GGC CAC TTG GCT CCT CTA TCT CCA
	Probes:	
B2M	Hs00187842	
MYC	Hs0153408	
CCNA1	Hs00171105	

### RNA interference

SKES1, A4573 and TC71 cells were plated in 96-well plates at a density of 5 × 10^4^ cells per well for assessing cell viability, or in 24-well plates at 1.5 × 10^5^ cells per well for RNA isolation purposes. On the following day, cells were transfected with 50 nM siRNA targeting human P300 (L-003486–00-0005) and CREBBP (L-003477–00-0005), or a non-targeting scrambled control siRNA (SMARTpool, Dharmacon), employing Lipofectamine RNAiMAX (Invitrogen) as the transfection agent. RNA was extracted 48 h after transfection, and the impact on cell viability was evaluated at both 48 and 72 h post-transfection.

Similarly, SKES1 cells were transfected with 50 nM siRNA targeting human P15 (L-003245–00-0005) under the same conditions. Downstream analyses were conducted after 24 h post-transfection and following 48 h of treatment with iP300w.

### Retrovirus infection

LMNB1 (GFP-LMNB1) and control GFP were overexpressed in SKES1 cells using retrovirus vectors pQCXIP-GFP and pQCXIP-GFP-myc-HsLMNB1, generously provided by Dr. Stephen A. Adam from Northwestern University [60]. The retroviruses were produced in 293 T packaging cells using pVSV-G (Clontech). Supernatant collected at 48 and 72 h post-infection, mixed with polybrene (10 µg/ml), was used to infect SKES1 cells. Selection was achieved by puromycin (2 µg/ml), and confirmation was conducted by FACS analysis of GFP-positive cells.

### β-galactosidase (β-Gal) cell staining

Cells were seeded in 96-well plates and at 50% confluence. The medium was removed, and cells were rinsed with PBS and fixed using 1 × fixative from the senescence β-Gal staining kit (9860, Cell Signaling Technology) for 15 min. After additional PBS washes, each well was stained with 50 μL of β-gal staining solution and incubated at 37 °C overnight. For senescence delay experiments, the staining period was shortened to 4 h for non-treated groups showing significant β-Gal positivity. Senescent cells were identified by β-Gal staining and quantified in at least three random fields.

### Annexin V staining

Apoptosis was assessed using the APC Annexin V Apoptosis Detection Kit with 7-AAD (Biolegend, 640930), strictly following the manufacturer's guidelines. After treatment, cells were harvested and labeled according to the kit's protocol. Analysis was performed on a BD FACSCanto II flow cytometer (Becton Dickinson, US), focusing on capturing mean fluorescence intensity (MFI) data. This data was then processed using FlowJo software (Tree Star, USA) to quantify apoptotic events.

### Bioinformatics

Paired-end Illumina sequencing reads were processed using TrimGalore (version 0.6.0), and transcript quantification was performed with Salmon (version 1.2.1) utilizing human Gencode annotations (version 34) and applying the GC-bias correction feature. Data importation into R (version 4.0.2) was achieved using tximeta (version 1.6.3). Differential expression analysis was conducted using DESeq2 (version 1.28.1). Visualization tools employed included ComplexHeatmap (version 2.4.3), clusterProfiler (version 3.16.1), UpsetR (version 1.4), and ggplot2 (version 3.3.2). Pathway enrichment analysis was performed using KEGG (https://www.genome.jp/kegg/), with figures generated by clusterProfiler (version 3.16.1). Clustering of patients was executed using NMF (version 0.27). Survival analysis utilized the Survival package (version 3.5) for single Cox models and glmnet (version 4.1) for Lasso Cox models. Lasso Cox regression involved tenfold cross-validation to identify the optimal value for the regularization parameter lambda (λ) [71], selected to minimize the partial likelihood deviance. The coefficient profiles of candidate genes were analyzed as a function of log2(lambda), with key predictors retained at the optimal λ value.

#### ChIP-Seq data were analyzed using IGV (version 2.16.2)

### Statistics

Statistical analyses were conducted using Graphpad Prism software, except as noted. The sample sizes were determined based on previous experience with similar assays to achieve sufficient statistical power. Variance within each group was comparable. Group comparisons were made using either one-way or two-way analysis of variance (ANOVA), with subsequent Tukey’s post-hoc tests for specific comparisons. Statistical significance was established at *p*-values less than or equal to 0.05.

## Supplementary Information

Supplementary Material 1: Figure S1: Effect of iP300w on ES cell lines in vitro and in vivo. (A) Comparison of cell viability reduction between ES cell lines (SKES1, CHLA9, CHLA25) and osteosarcoma cell lines (U2OS, 143B, SJSA-1, G292) after 48 hours of treatment with 0.1 and 1 μM iP300w. Data are presented as mean ± SEM; *p<* 0.05 by two-way ANOVA (*n* = 3). (B) Representative Ki-67 (red) staining in ES cell lines (TC71, A4573, A673) after 48 hours of 1 μM iP300w treatment. Nuclei are stained with blue (DAPI). (C) RT-qPCR analysis shows changes in EWS::FLI1 target genes in ES cells following 24 hours of treatment with 1μM of iP300w. The data represent mean ± SEM; *p<0.05 by two-way ANOVA (*n*=6). (D) Gene expression changes (RNA-Seq) in ES cells 4 hours post-iP300w treatment. Data represent count, *p<0.05 by t-test (*n*=2). (E) RT-qPCR analysis of EWS::FLI1 target genes in SKES1 xenografts following 14 days of iP300w treatment (5.6 mg/kg daily). Data are presented as mean ± SEM and analyzed using a t-test; *p< 0.01, **p< 0.001, ***p< 0.0001 (*n* = 6).

Supplementary Material 2: Figure S2: Dynamic tumor ES tumor development and toxicity assessment of iP300w. (A) Tumor volume in mm³ measured at day 14, day 17, and day 20 in the iP300w treatment (4.2 mg/kg daily). Data are analyzed using two-way ANOVA and presented as mean ± SEM; *p<0.05 (*n*=5). (B) Line graph showing body weight measurements for control and iP300w-treated (4.2 mg/kg daily) groups over a 20-day period. Data were analyzed using two-way ANOVA and are presented as mean ± SEM; no significant differences were observed between the groups. (C) Bar graphs showing serum levels of BUN, creatinine, Na, K, osmolality, ALP, ALT, and glucose in treated mice compared to controls. Data are analyzed using t-test and presented as mean ± SEM; **p<0.01 (*n*=6). (D) Representative H&E images showing liver and kidney tissues from treated and control mice.

Supplementary Material 3: Figure S3: PCA clustering of iP300w treated ES cell lines. (A)PCA clustering of transcriptional profiles (RNA-Seq) from ES cancer cell lines (A4573, A673, TC71 and SKES1) treated with iP300w for 4 hours.PCA clustering of ES and non ES cancer cell line treated with iP300w for 4 hours. (B)PCA clustering of ES and non ES cancer cell line (143B, A204, Fuji, G292, HOS, Kitra-SRS, MG-63, SJSA-1, T47, U2OS) treated with iP300w for 4 hours. (C) ATP assay comparing viability of SKES1 ES cell line to other cancer cell lines (FUJI, A204, T47D, HOS) after 24 hours of iP300w treatment. Data were analyzed using two-way ANOVA and are presented as mean ± SEM; *p<* 0.05 (*n* = 3).

Supplementary Material 4: Figure S4: Lasso Cox regression analysis for identifying survival-associated genes. The upper panel shows the coefficient profiles of selected genes as a function of the regularization parameter (log2(lambda)) [71]. As lambda increases, the coefficients of more genes are shrunk towards zero, with a subset of genes (TSKU, IKZF2, NTN4, IL20RB, SMAD9, NDST4, NKX2-2, ZBTB16) remaining significant at the optimal lambda value (λ = 0.11). The lower panel presents the Partial Likelihood Deviance as a function of log2(lambda), where the red dotted line indicates the optimal lambda value (λ = 0.11) selected based on the minimum deviance. Error bars represent the standard errors of the deviance.

Supplementary Material 5: Figure S5: P300/CBP inhibition triggers senescence in ES cell lines with (A4573 and CHLA9) and without (TC71 and CHLA25) functional P53. (A) β-Gal and H&E staining of TC71 cells following iP300w (1 µM) treatment for 24 and 72 hours. (B) β-Gal staining in CHLA25, A4573 and CHLA9 cells after 72 hours iP300w treatment. (C) Gene expression analysis reveals changes in proliferation and senescence markers in TC71, CHLA25, A4573, and CHLA9 cells following 1, 3, and 7 days of treatment with 1 μM iP300w. Data were normalized to B2M and are presented as log2 fold change compared to the control group. Data were analyzed using two-way ANOVA and are presented as mean ± SEM; *p<* 0.05. Note that LMNB1 suppression occurs in all cell lines, while P15 induction is observed only in ES cell lines with reported non-functional P53 (TC71 and CHLA25). (D) Immunostaining for LMNB1 and P15 in iP300w treated cells after 72 hours of incubation correlated with the gene expression analyses.

Supplementary Material 6: Figure S6: P300/CBP inhibition triggers senescence in ES cells line harboring EWS::ERG translocation. (A) ATP assay on EWS::FLI1 cell line (SKES1) and EWS::ERG cell lines (CADO-ES1 and COGE352) after 48 hours of treatment with 1 μM iP300w. The data represent mean ± SEM; *p<0.05, by two-way ANOVA (*n*=6). (B) Immunostaining shows changes in Ki67 (red) expression after 72 hours iP300w treatment. (C) Gene expression analysis shows changes in proliferation and senescence markers in CADO-ES1 cells following 1 day, 3 days, and 7 days of treatment with 1μM iP300w. Data were normalized to B2M and are presented as log2 fold change compared to the control group. Data are analyzed using two-way ANOVA and presented as mean ± SEM; * *p<*0.05. (D) β-Gal staining in CADO-ES1 cells following 72 hours of P300/CBP inhibition. (E) Immunostaining for LMNB1 and P15 in CADO-ES1 cells after 72 hours iP300w treatment. (F) Gene expression analysis shows changes in proliferation and senescence markers in COGE352 cells following 1 day, 3 days, and 7 days of treatment with 1μM iP300w. Data were normalized to B2M and are presented as log2 fold change compared to the control group. Data are analyzed using two-way ANOVA and presented as mean ± SEM; * *p<*0.05. (G) β-Gal staining in COGE352 cells at 72 hours of treatment. (H) Immunostaining for LMNB1 and P15 in 72 hours iP300w treated COGE352 cells.

Supplementary Material 7: Figure S7: Senolytic screening, cell viability, and apoptosis in ES cell lines treated with iP300w. (A) RNA-Seq analysis of senolytic targets following 4 hours of treatment of SKES1 and TC71 with 1 µM iP300w. Data are analyzed using two-way ANOVA and presented as mean ± SEM; * *p<*0.05, (*n*=2). (B) ATP assays show SKES1 cell viability at different concentrations (0.01 μM, 0.1 μM, 1 μM, 10 μM, 20 μM, and 50 μM) of senolytics, used alone or combined with 1 μM iP300w, with data collected after 72 hours of treatment. Data are analyzed using two-way ANOVA and presented as mean ± SEM; * *p<*0.05 (*n*=4). (C) ATP assays show TC71 cell viability at different concentrations (0.01 μM, 0.1 μM, 1 μM, 10 μM, 20 μM, and 50 μM) of senolytics, used alone or combined with 1 μM iP300w, with data collected after 72 hours of treatment. Data are analyzed using two-way ANOVA and presented as mean ± SEM; * *p<*0.05 (*n*=4). (D) ATP assays show TC71 cell viability with different senolytics (1 μM) used alone or combined with 1 μM iP300w for 72 hours. Data is presented as mean ± SEM; two-way ANOVA, **p<0.001, ****p<0.0001 (*n*=4). (E) Synergy scores between iP300w and various senolytics (Dasatinib, Fisetin, Navitoclax, and Quercetin) were calculated using the Highest Single Agent (HSA) model via SynergyFinder Plus. The 3D plots display synergy scores across different concentrations of senolytics (X-axis: 0 μM, 0.01 μM, 0.1 μM, 1 μM, 10 μM, 50 μM) combined with iP300w (Y-axis: 0 μM, 1 μM). The Z-axis represents the synergy score, with positive values indicating synergy and negative values indicating antagonism. (F) FACS analyses for Annexin V positive SKES1 cells following 72 hours of treatment with iP300w (1 μM), Dasatinib (1 μM), Dasatinib+iP300w, and Doxorubicin (1 μM). (G) Quantification of Annexin V staining. Data are presented as the percentage of positive cells. The data represent mean ± SEM; *p<0.05, ****p<0.0001 by one-way ANOVA (*n*=3).

Supplementary Material 8: Figure S8: EWS::FLI1 and EWS::ERG bind to regulatory elements of senescence-related genes. (A) ChIP-Seq analysis explores EWS::FLI1 binding sites, H3K27ac and P300 in the regulatory elements of LMNB1, P15, P16, TP53 and P21 in SKNMC before and after EWS::FLI1 knockdown. (B) ChIP-Seq data shows EWS::FLI1 and acH3K27 binding sites in LMNB1, P15, P16, TP53 and P21 in MSC overexpressing EWS::FLI1. (C) ChIP-Seq analysis shows binding sites in the regulatory elements of LMNB1, P15, P16, TP53, and P21 for EWS::FLI1 in SKES1 and EWS::ERG in TC32. (D) Transcriptional levels (RNA-Seq) of P16, TP53 and P21 following EWS::FLI1 knockdown (96 hours). (E) P16, TP53 and P21 expression (RNA-Seq) in MSC overexpressing EWS::FLI1 and GFP control cells. Data are presented as mean expression levels ± SEM; *p<*0.05 by t-test (*n*=4).

Supplementary Material 9: Figure S9: LMNB1 overexpression and P15 knockdown in senescence-induced SKES1 cells. (A) RT-qPCR analysis shows constitutive LMNB1 overexpression and P15 knockdown in SKES1 cells. LB1 refers to SKES1 cells that constitutively overexpress LMNB1 from a viral construct, while siP15 denotes SKES1 cells with P15 knocked down. LMNB1+siP15 refers to the condition where LMNB1 is expressed and P15 is knocked down for 48 hours. Cells were treated with  iP300w (1 µM) for 48 hours. The data represent mean ± SEM, analyzed using two-way ANOVA; *p<* 0.05 (*n*=3). (B) FACS analyses of Annexin V staining for SKES1 cells in the condition described above. (C) Quantification of percent of Annexin V positive cells. Data are analyzed using one-way ANOVA and presented as mean ± SEM; ***p<0.001, ****p<0.0001.

Supplementary Material 10: Figure S10: Original images of Western blots. Raw images of western blots

## Data Availability

Sequencing reads and processed data have been deposited into GEO, SRA. The accession number: PRJNA1161150.

## Data availability

No datasets were generated or analysed during the current study.
