# Supplementary figures and images for: Pharmacological targeting of P300/CBP reveals EWS::FLI1-mediated senescence evasion in Ewing sarcoma
Source: Mol Cancer. 2024 Oct 5;23:222. doi: 10.1186/s12943-024-02115-7 (PMC11453018; doi:10.1186/s12943-024-02115-7)

Supplementary Fig. 1

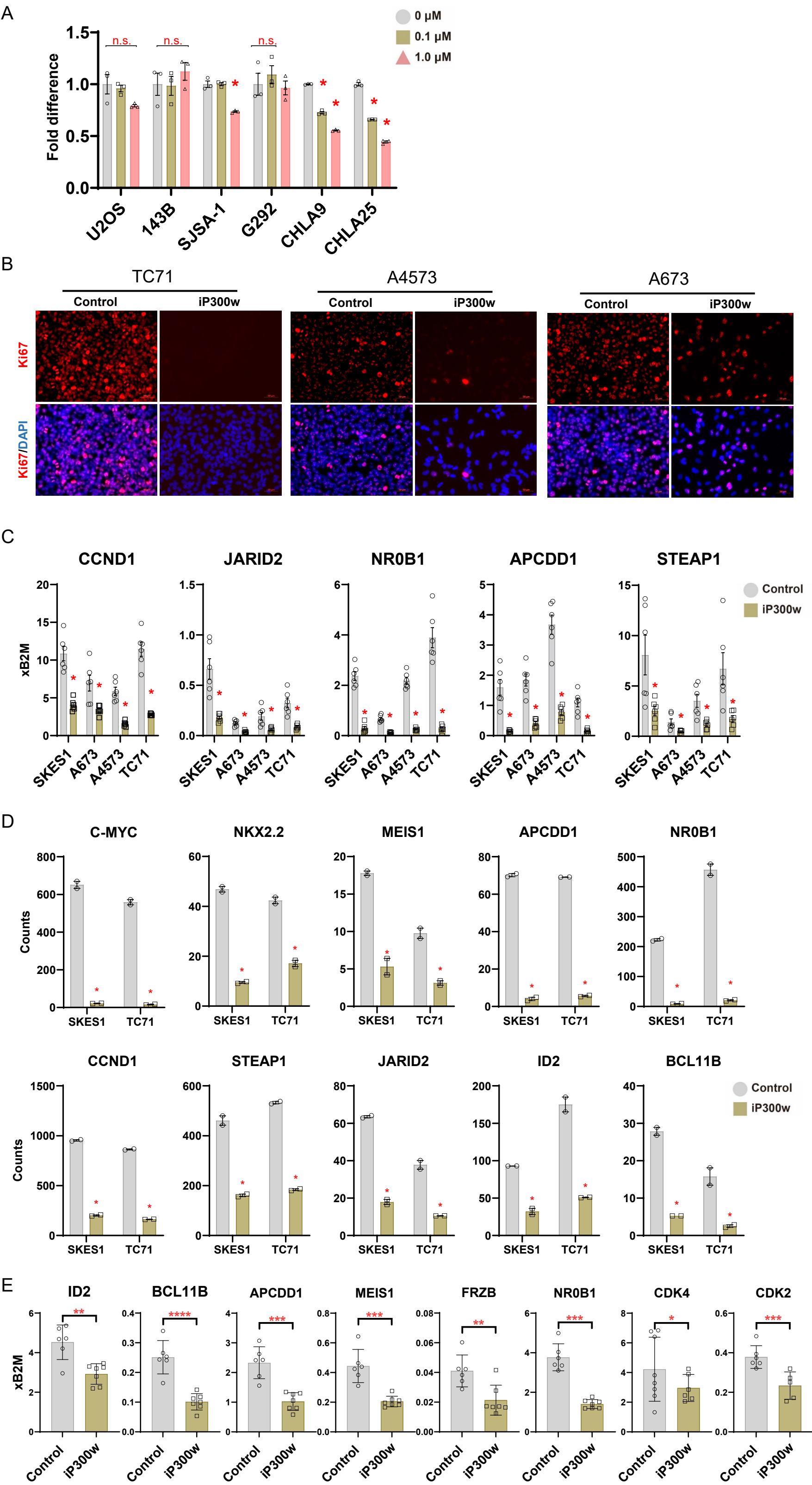

Supplement: Supplementary file 1 — Supplementary Material 1: Figure S1: Effect of iP300w on ES cell lines in vitro and in vivo. (A) Comparison of cell viability reduction between ES cell lines (SKES1, CHLA9, CHLA25) and osteosarcoma cell lines (U2OS, 143B, SJSA-1, G292) after 48 hours of treatment with 0.1 and 1 μM iP300w. Data are presented as mean ± SEM; p< 0.05 by two-way ANOVA (n = 3). (B) Representative Ki-67 (red) staining in ES cell lines (TC71, A4573, A673) after 48 hours of 1 μM iP300w treatment. Nuclei are stained with blue (DAPI). (C) RT-qPCR analysis shows changes in EWS::FLI1 target genes in ES cells following 24 hours of treatment with 1μM of iP300w. The data represent mean ± SEM; *p<0.05 by two-way ANOVA (n=6). (D) Gene expression changes (RNA-Seq) in ES cells 4 hours post-iP300w treatment. Data represent count, *p<0.05 by t-test (n=2). (E) RT-qPCR analysis of EWS::FLI1 target genes in SKES1 xenografts following 14 days of iP300w treatment (5.6 mg/kg daily). Data are presented as mean ± SEM and analyzed using a t-test; *p< 0.01, **p< 0.001, ***p< 0.0001 (n = 6). [file 12943_2024_2115_MOESM1_ESM.pdf]

Supplementary Figure 2

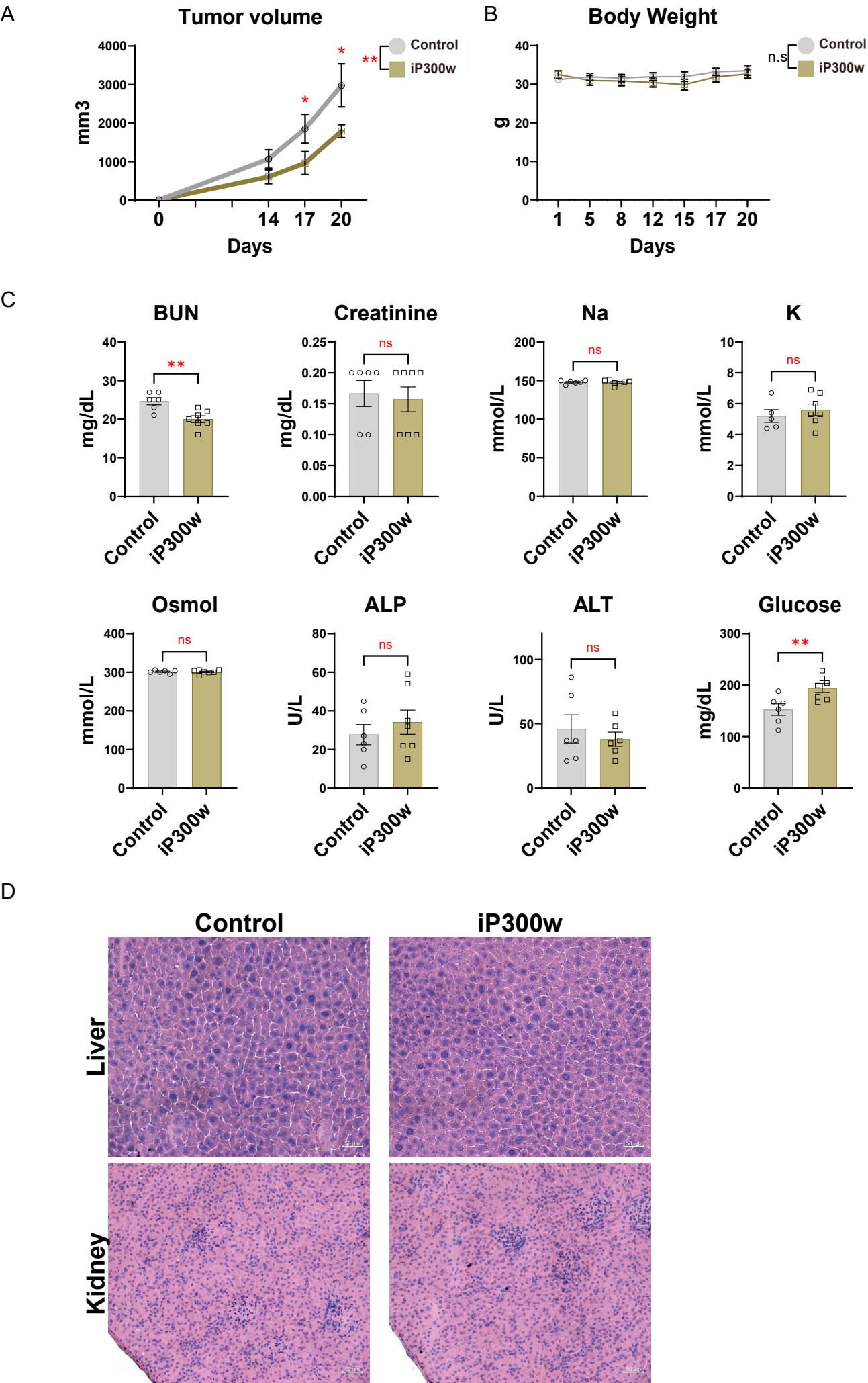

Supplement: Supplementary file 2 — Supplementary Material 2: Figure S2: Dynamic tumor ES tumor development and toxicity assessment of iP300w. (A) Tumor volume in mm³ measured at day 14, day 17, and day 20 in the iP300w treatment (4.2 mg/kg daily). Data are analyzed using two-way ANOVA and presented as mean ± SEM; *p<0.05 (n=5). (B) Line graph showing body weight measurements for control and iP300w-treated (4.2 mg/kg daily) groups over a 20-day period. Data were analyzed using two-way ANOVA and are presented as mean ± SEM; no significant differences were observed between the groups. (C) Bar graphs showing serum levels of BUN, creatinine, Na, K, osmolality, ALP, ALT, and glucose in treated mice compared to controls. Data are analyzed using t-test and presented as mean ± SEM; **p<0.01 (n=6). (D) Representative H&E images showing liver and kidney tissues from treated and control mice. [file 12943_2024_2115_MOESM2_ESM.pdf]

Supplementary Figure 3

A

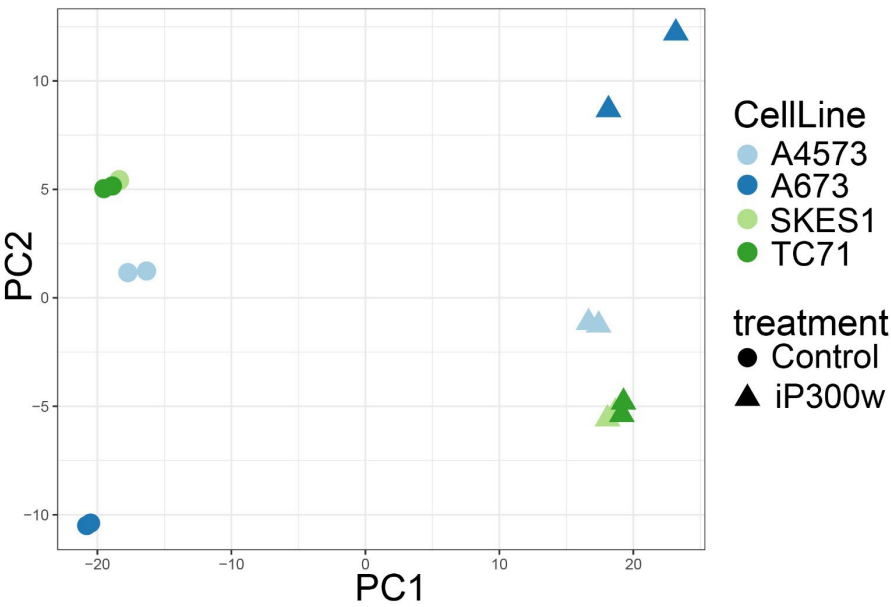

B

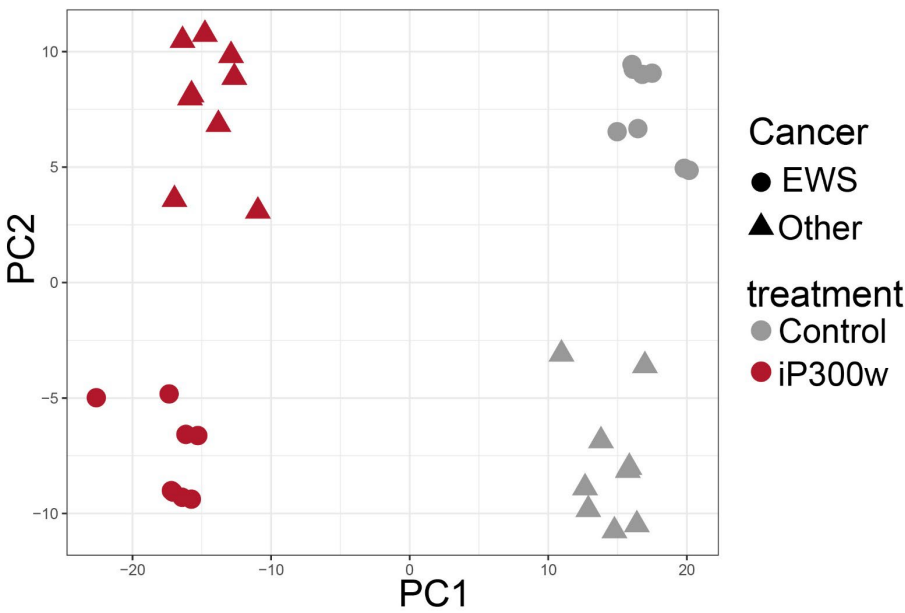

C

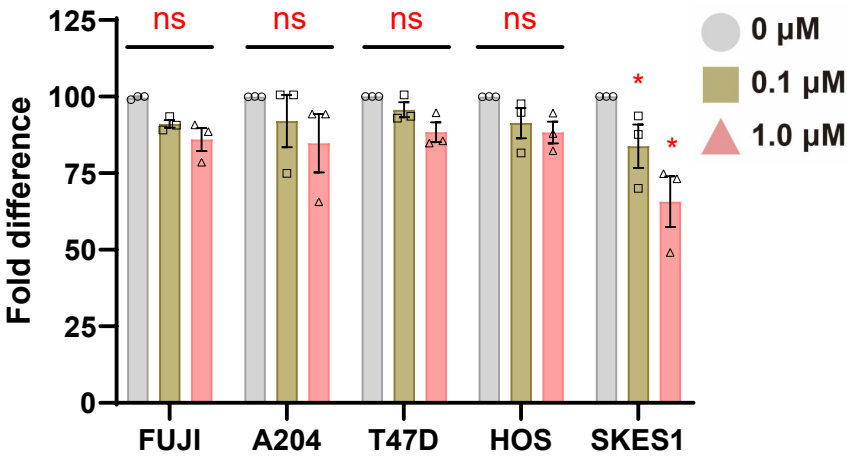

Supplement: Supplementary file 3 — Supplementary Material 3: Figure S3: PCA clustering of iP300w treated ES cell lines. (A)PCA clustering of transcriptional profiles (RNA-Seq) from ES cancer cell lines (A4573, A673, TC71 and SKES1) treated with iP300w for 4 hours.PCA clustering of ES and non ES cancer cell line treated with iP300w for 4 hours. (B)PCA clustering of ES and non ES cancer cell line (143B, A204, Fuji, G292, HOS, Kitra-SRS, MG-63, SJSA-1, T47, U2OS) treated with iP300w for 4 hours. (C) ATP assay comparing viability of SKES1 ES cell line to other cancer cell lines (FUJI, A204, T47D, HOS) after 24 hours of iP300w treatment. Data were analyzed using two-way ANOVA and are presented as mean ± SEM; p< 0.05 (n = 3). [file 12943_2024_2115_MOESM3_ESM.pdf]

Supplementary Figure 4

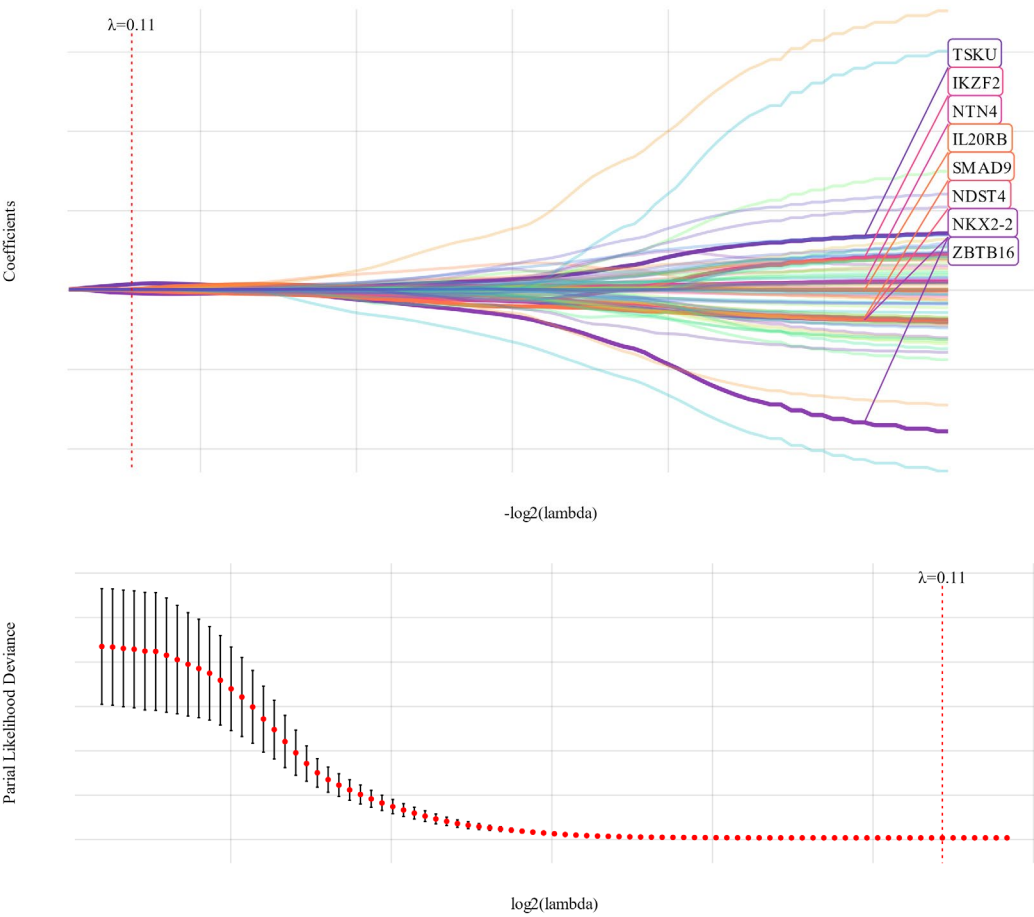

Supplement: Supplementary file 4 — Supplementary Material 4: Figure S4: Lasso Cox regression analysis for identifying survival-associated genes. The upper panel shows the coefficient profiles of selected genes as a function of the regularization parameter (log2(lambda)) [71]. As lambda increases, the coefficients of more genes are shrunk towards zero, with a subset of genes (TSKU, IKZF2, NTN4, IL20RB, SMAD9, NDST4, NKX2-2, ZBTB16) remaining significant at the optimal lambda value (λ = 0.11). The lower panel presents the Partial Likelihood Deviance as a function of log2(lambda), where the red dotted line indicates the optimal lambda value (λ = 0.11) selected based on the minimum deviance. Error bars represent the standard errors of the deviance. [file 12943_2024_2115_MOESM4_ESM.pdf]

Supplementary Figure 5

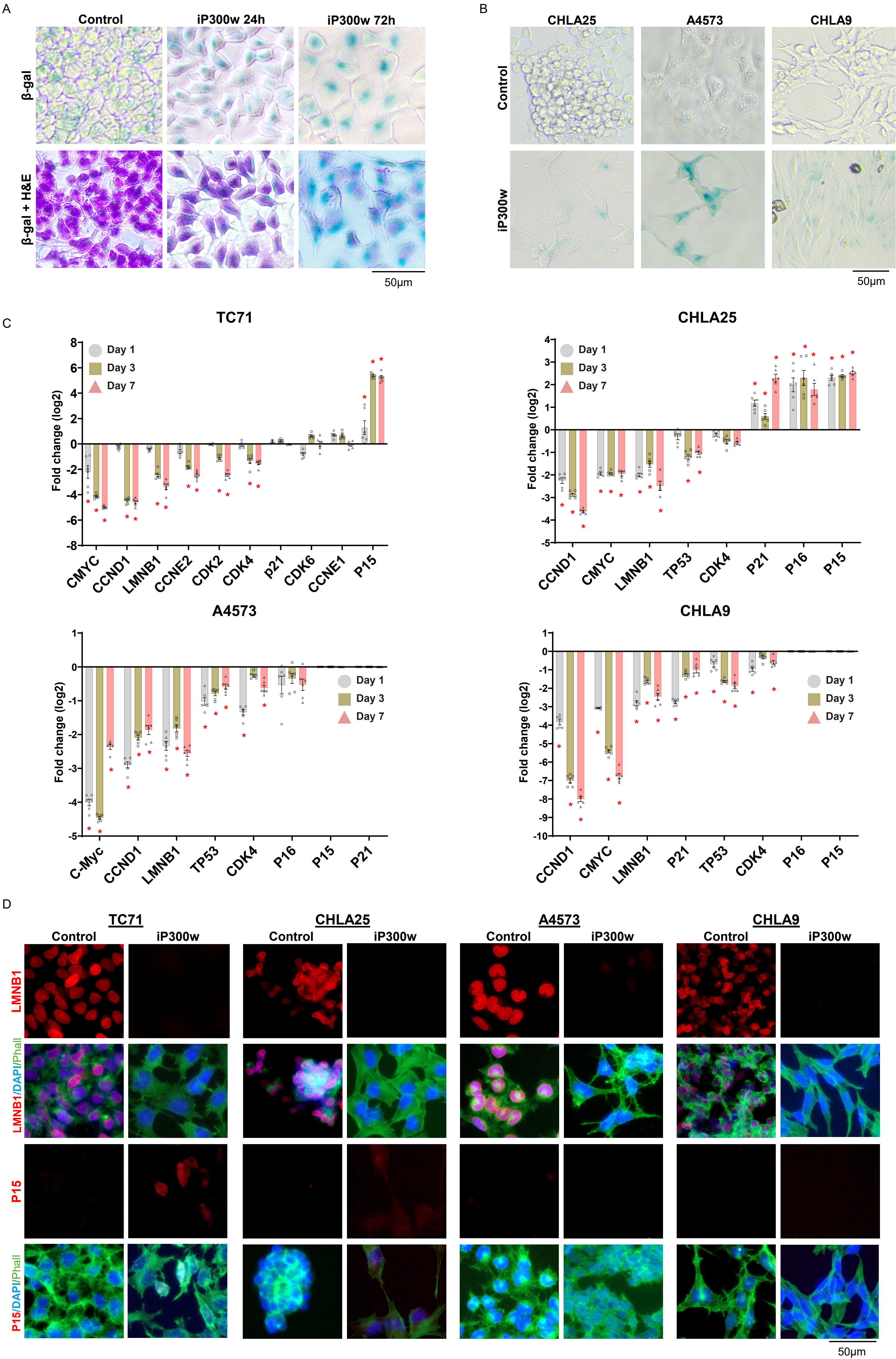

Supplement: Supplementary file 5 — Supplementary Material 5: Figure S5: P300/CBP inhibition triggers senescence in ES cell lines with (A4573 and CHLA9) and without (TC71 and CHLA25) functional P53. (A) β-Gal and H&E staining of TC71 cells following iP300w (1 µM) treatment for 24 and 72 hours. (B) β-Gal staining in CHLA25, A4573 and CHLA9 cells after 72 hours iP300w treatment. (C) Gene expression analysis reveals changes in proliferation and senescence markers in TC71, CHLA25, A4573, and CHLA9 cells following 1, 3, and 7 days of treatment with 1 μM iP300w. Data were normalized to B2M and are presented as log2 fold change compared to the control group. Data were analyzed using two-way ANOVA and are presented as mean ± SEM; p< 0.05. Note that LMNB1 suppression occurs in all cell lines, while P15 induction is observed only in ES cell lines with reported non-functional P53 (TC71 and CHLA25). (D) Immunostaining for LMNB1 and P15 in iP300w treated cells after 72 hours of incubation correlated with the gene expression analyses. [file 12943_2024_2115_MOESM5_ESM.pdf]

Supplementary Figure 6

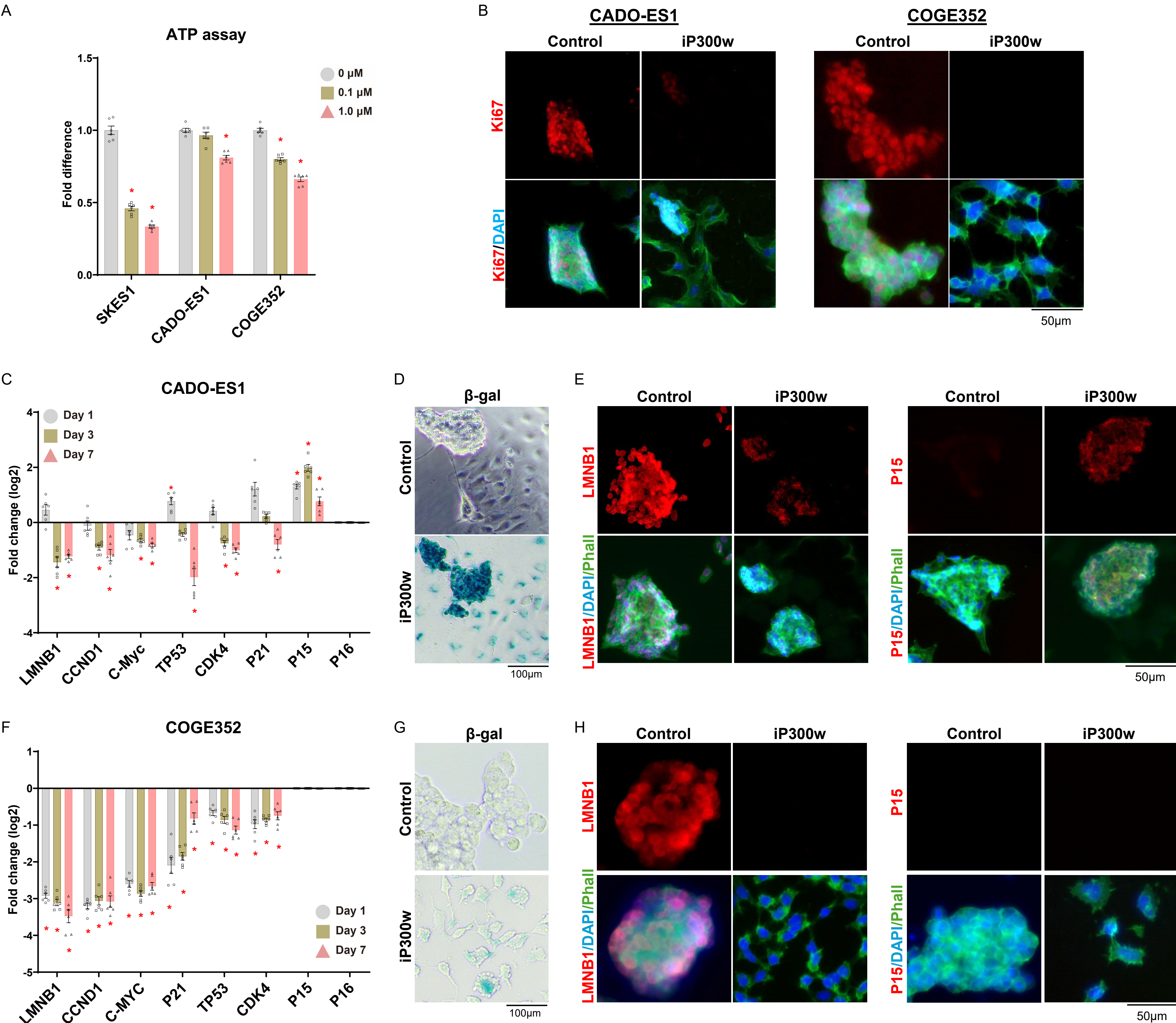

Supplement: Supplementary file 6 — Supplementary Material 6: Figure S6: P300/CBP inhibition triggers senescence in ES cells line harboring EWS::ERG translocation. (A) ATP assay on EWS::FLI1 cell line (SKES1) and EWS::ERG cell lines (CADO-ES1 and COGE352) after 48 hours of treatment with 1 μM iP300w. The data represent mean ± SEM; *p<0.05, by two-way ANOVA (n=6). (B) Immunostaining shows changes in Ki67 (red) expression after 72 hours iP300w treatment. (C) Gene expression analysis shows changes in proliferation and senescence markers in CADO-ES1 cells following 1 day, 3 days, and 7 days of treatment with 1μM iP300w. Data were normalized to B2M and are presented as log2 fold change compared to the control group. Data are analyzed using two-way ANOVA and presented as mean ± SEM; * p<0.05. (D) β-Gal staining in CADO-ES1 cells following 72 hours of P300/CBP inhibition. (E) Immunostaining for LMNB1 and P15 in CADO-ES1 cells after 72 hours iP300w treatment. (F) Gene expression analysis shows changes in proliferation and senescence markers in COGE352 cells following 1 day, 3 days, and 7 days of treatment with 1μM iP300w. Data were normalized to B2M and are presented as log2 fold change compared to the control group. Data are analyzed using two-way ANOVA and presented as mean ± SEM; * p<0.05. (G) β-Gal staining in COGE352 cells at 72 hours of treatment. (H) Immunostaining for LMNB1 and P15 in 72 hours iP300w treated COGE352 cells. [file 12943_2024_2115_MOESM6_ESM.pdf]

Supplementary Figure 7

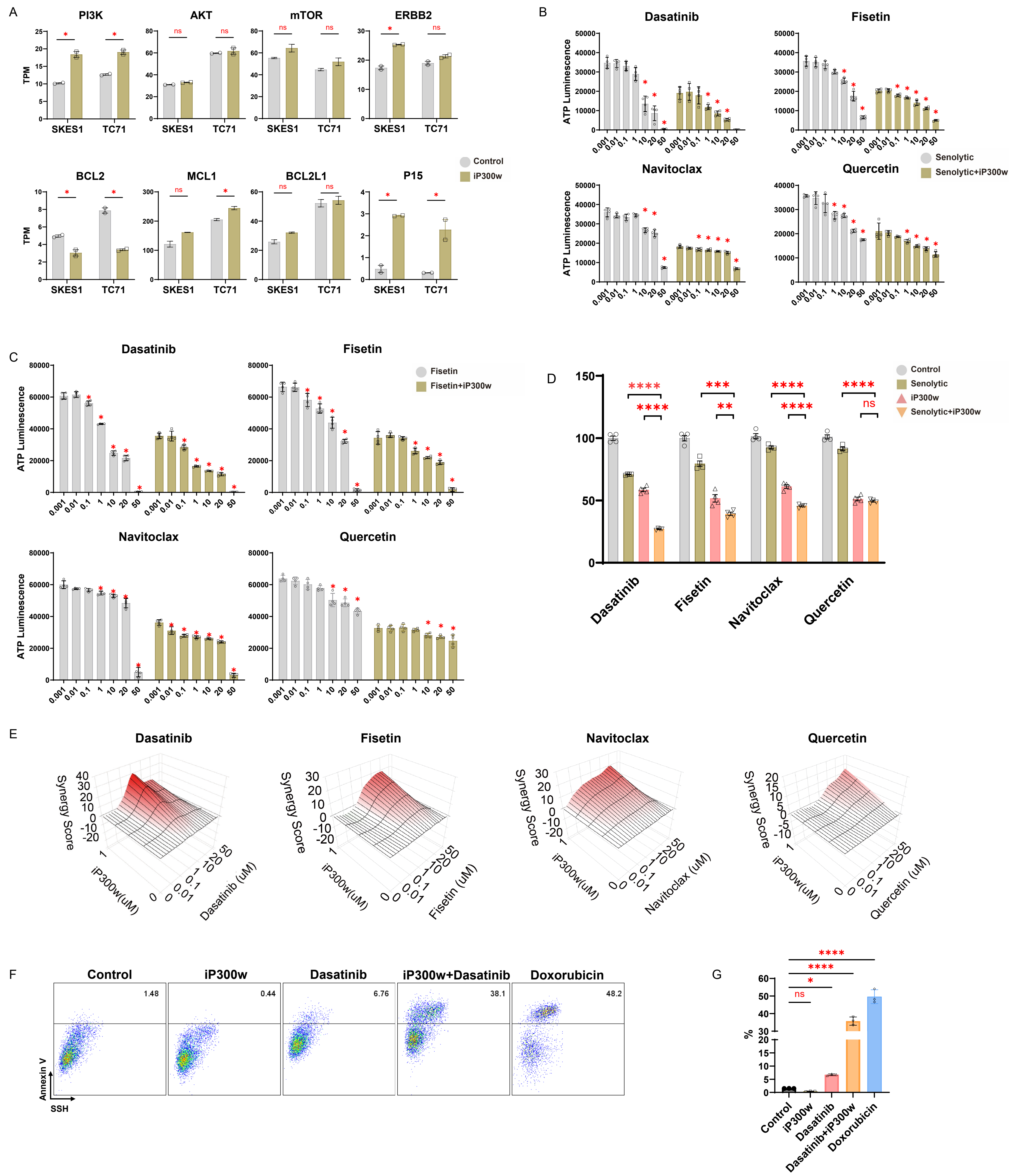

Supplement: Supplementary file 7 — Supplementary Material 7: Figure S7: Senolytic screening, cell viability, and apoptosis in ES cell lines treated with iP300w. (A) RNA-Seq analysis of senolytic targets following 4 hours of treatment of SKES1 and TC71 with 1 µM iP300w. Data are analyzed using two-way ANOVA and presented as mean ± SEM; * p<0.05, (n=2). (B) ATP assays show SKES1 cell viability at different concentrations (0.01 μM, 0.1 μM, 1 μM, 10 μM, 20 μM, and 50 μM) of senolytics, used alone or combined with 1 μM iP300w, with data collected after 72 hours of treatment. Data are analyzed using two-way ANOVA and presented as mean ± SEM; * p<0.05 (n=4). (C) ATP assays show TC71 cell viability at different concentrations (0.01 μM, 0.1 μM, 1 μM, 10 μM, 20 μM, and 50 μM) of senolytics, used alone or combined with 1 μM iP300w, with data collected after 72 hours of treatment. Data are analyzed using two-way ANOVA and presented as mean ± SEM; * p<0.05 (n=4). (D) ATP assays show TC71 cell viability with different senolytics (1 μM) used alone or combined with 1 μM iP300w for 72 hours. Data is presented as mean ± SEM; two-way ANOVA, **p<0.001, ****p<0.0001 (n=4). (E) Synergy scores between iP300w and various senolytics (Dasatinib, Fisetin, Navitoclax, and Quercetin) were calculated using the Highest Single Agent (HSA) model via SynergyFinder Plus. The 3D plots display synergy scores across different concentrations of senolytics (X-axis: 0 μM, 0.01 μM, 0.1 μM, 1 μM, 10 μM, 50 μM) combined with iP300w (Y-axis: 0 μM, 1 μM). The Z-axis represents the synergy score, with positive values indicating synergy and negative values indicating antagonism. (F) FACS analyses for Annexin V positive SKES1 cells following 72 hours of treatment with iP300w (1 μM), Dasatinib (1 μM), Dasatinib+iP300w, and Doxorubicin (1 μM). (G) Quantification of Annexin V staining. Data are presented as the percentage of positive cells. The data represent mean ± SEM; *p<0.05, ****p<0.0001 by one-way ANOVA (n=3). [file 12943_2024_2115_MOESM7_ESM.pdf]

Supplementary Figure 9

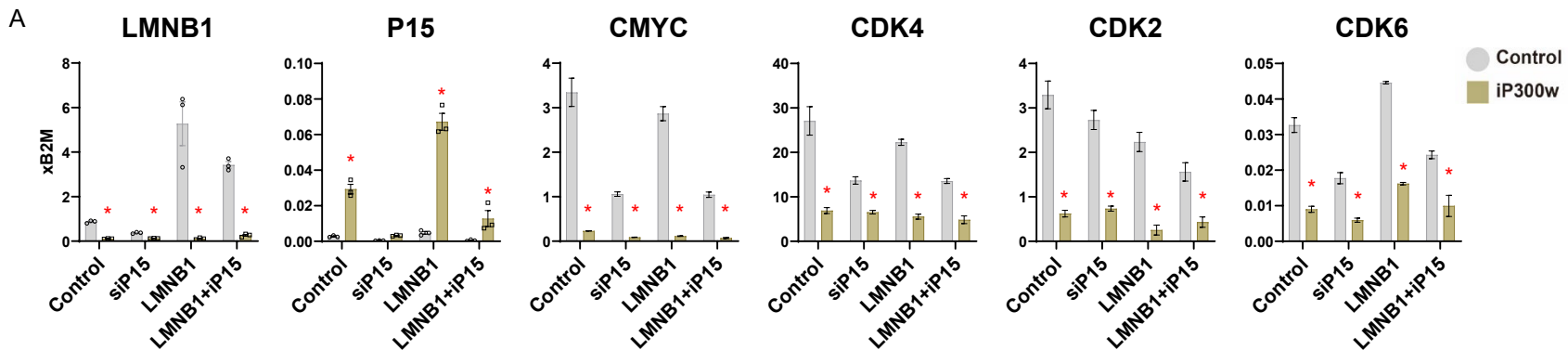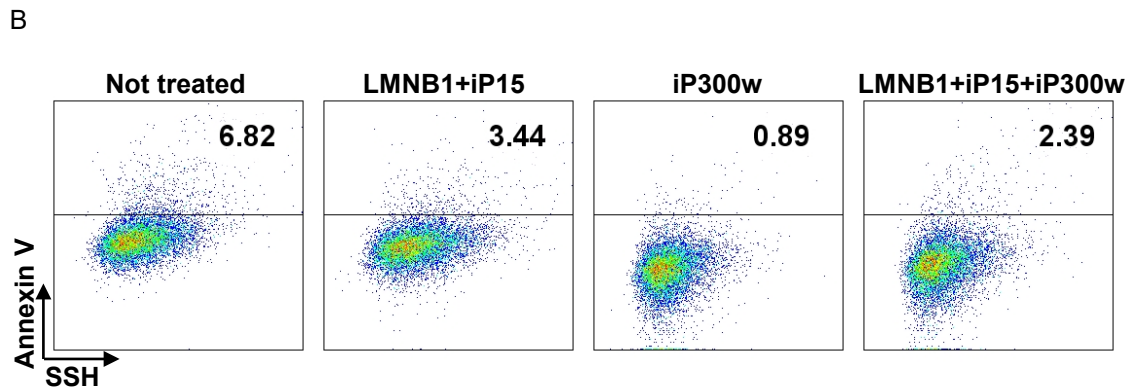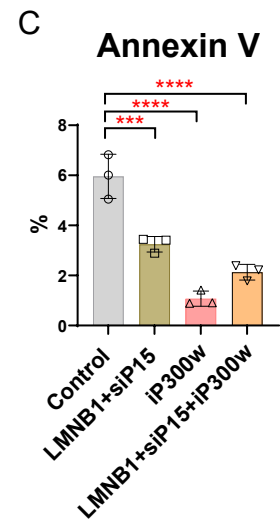

Supplement: Supplementary file 9 — Supplementary Material 9: Figure S9: LMNB1 overexpression and P15 knockdown in senescence-induced SKES1 cells. (A) RT-qPCR analysis shows constitutive LMNB1 overexpression and P15 knockdown in SKES1 cells. LB1 refers to SKES1 cells that constitutively overexpress LMNB1 from a viral construct, while siP15 denotes SKES1 cells with P15 knocked down. LMNB1+siP15 refers to the condition where LMNB1 is expressed and P15 is knocked down for 48 hours. Cells were treated with iP300w (1 µM) for 48 hours. The data represent mean ± SEM, analyzed using two-way ANOVA; p< 0.05 (n=3). (B) FACS analyses of Annexin V staining for SKES1 cells in the condition described above. (C) Quantification of percent of Annexin V positive cells. Data are analyzed using one-way ANOVA and presented as mean ± SEM; ***p<0.001, ****p<0.0001. [file 12943_2024_2115_MOESM9_ESM.pdf]

Supplementary Figure 10

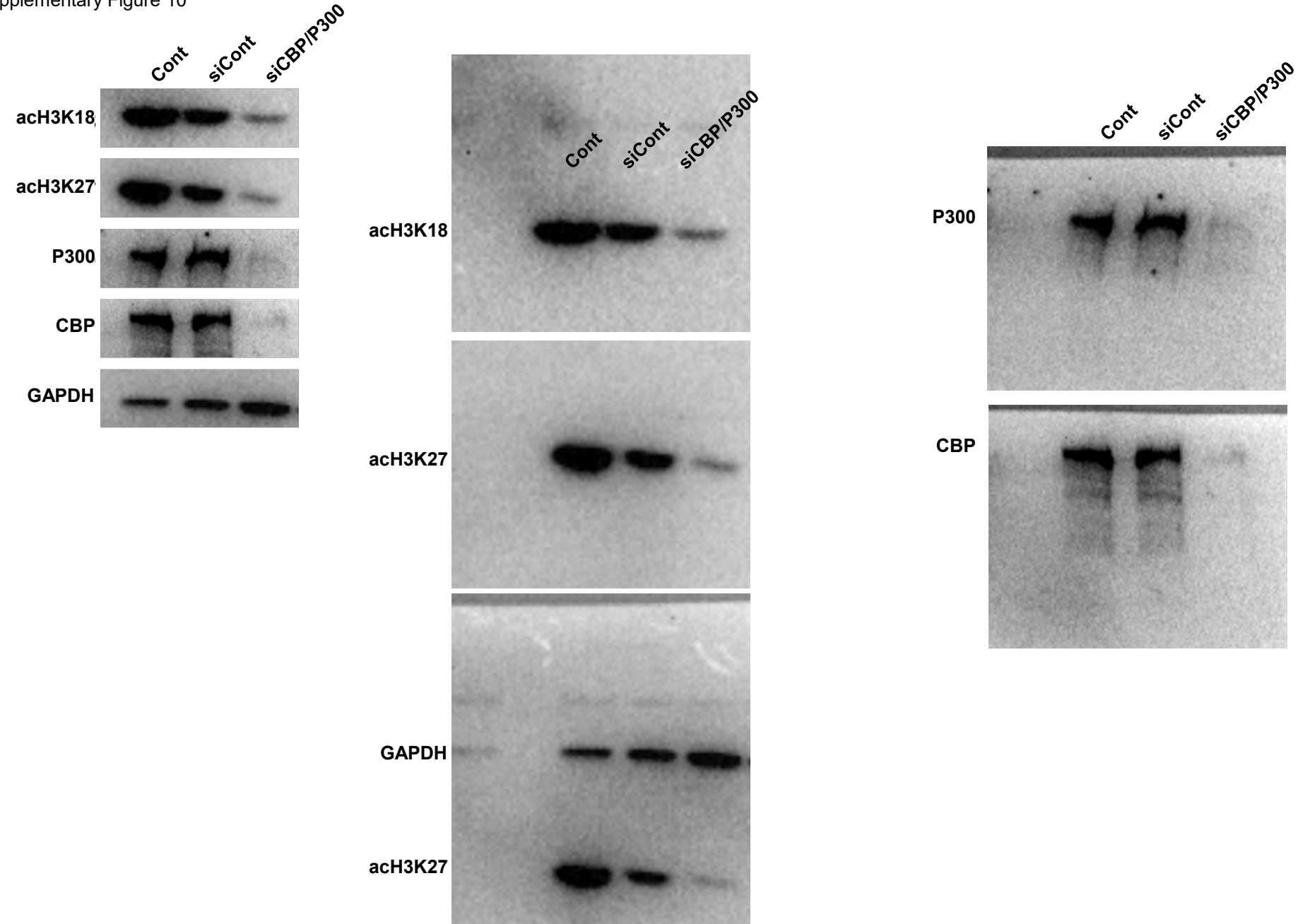

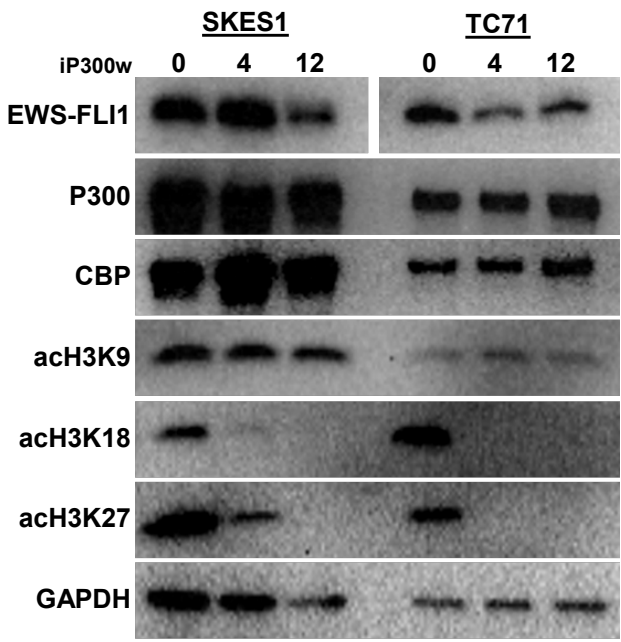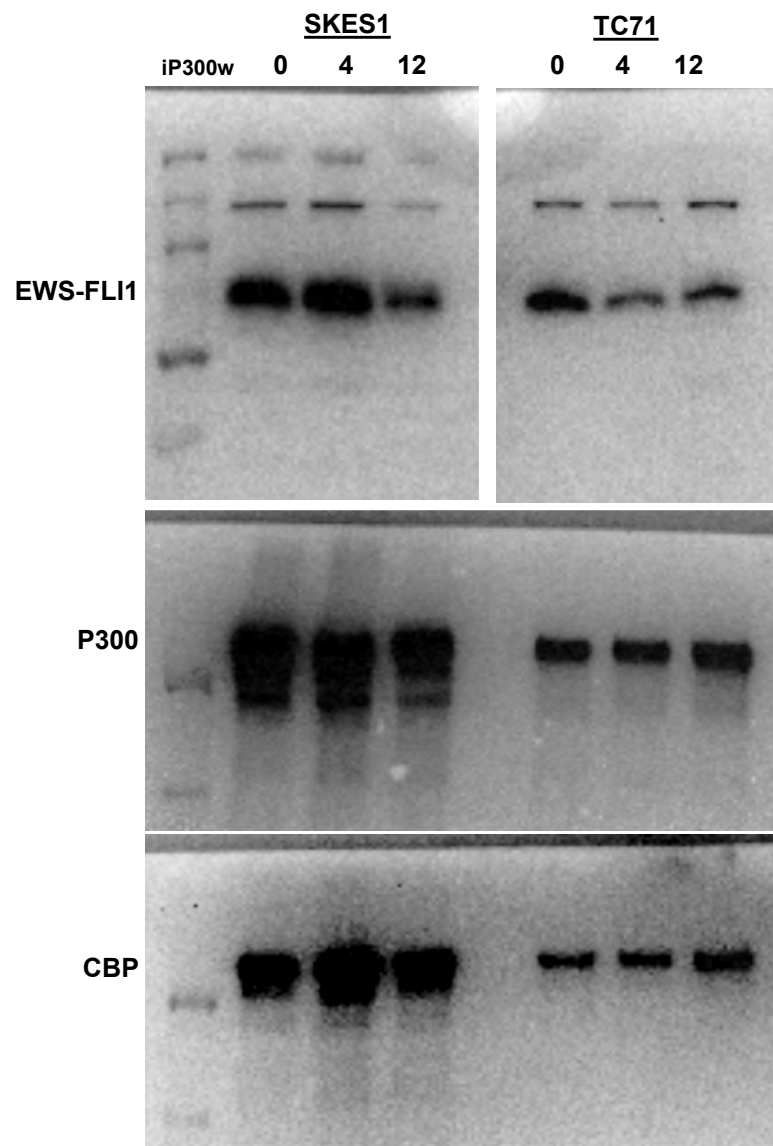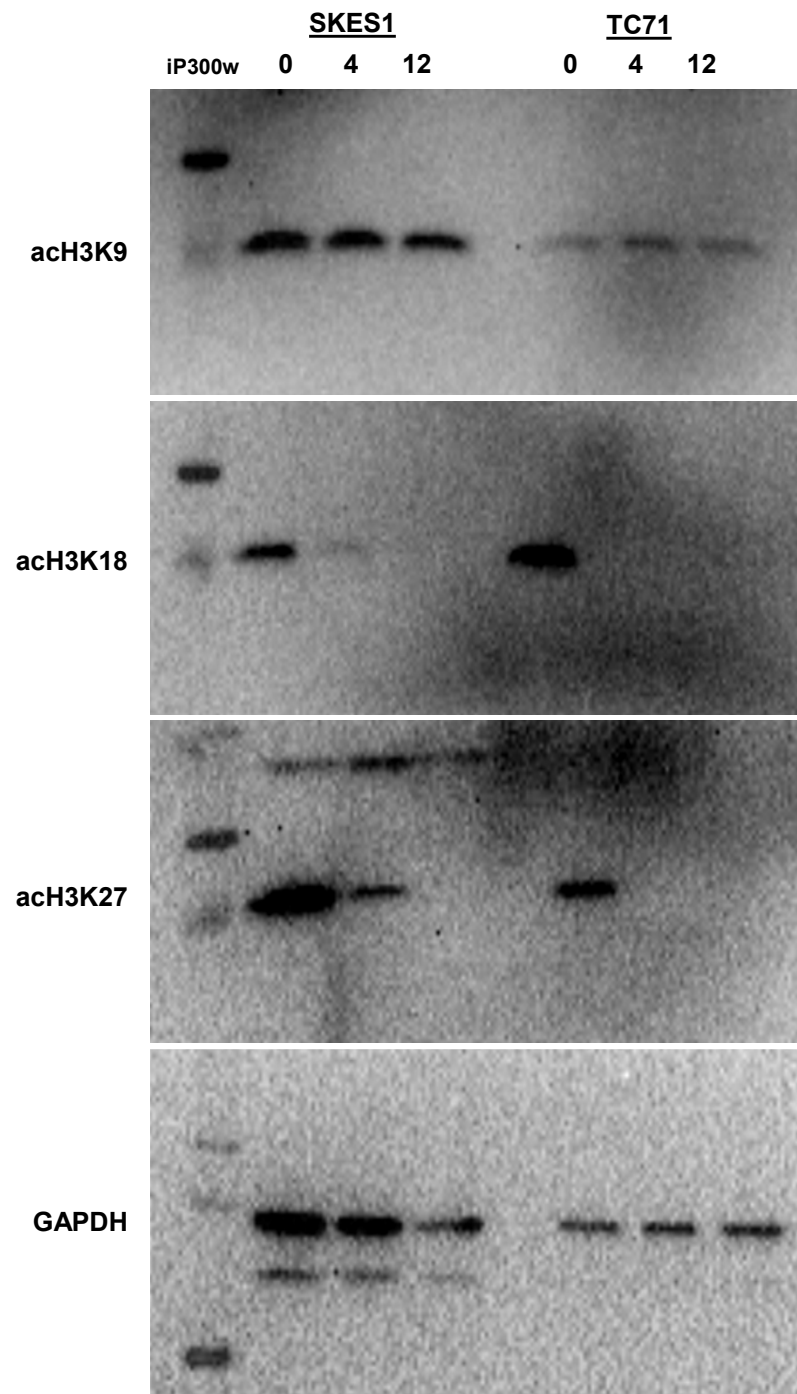

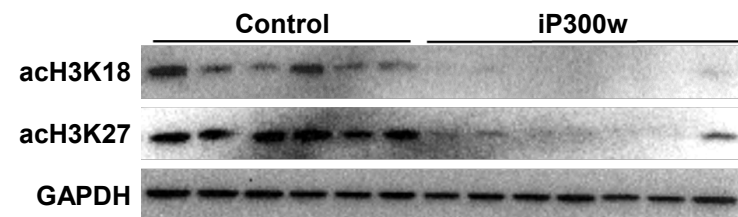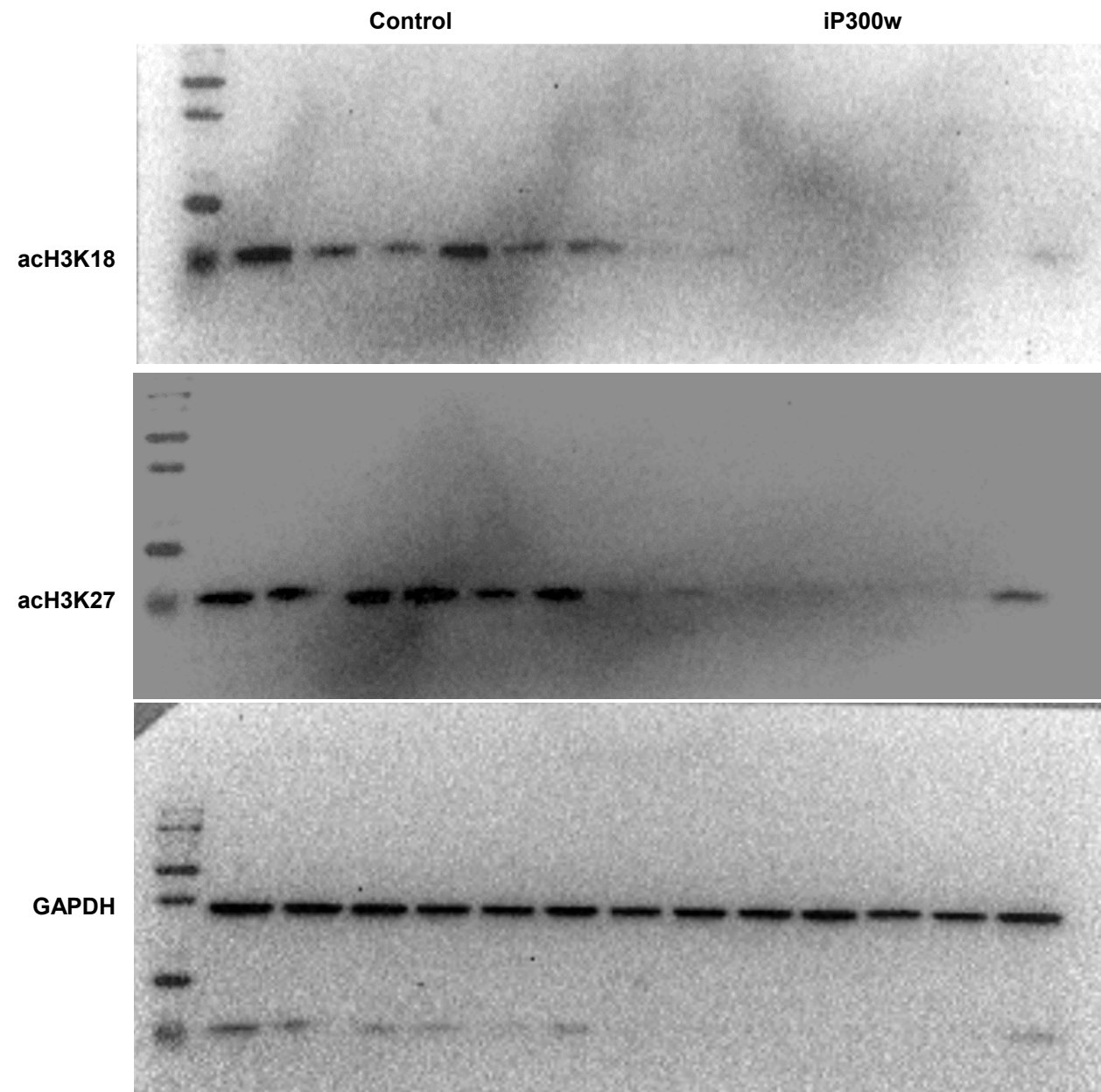

Supplement: Supplementary file 10 — Supplementary Material 10: Figure S10: Original images of Western blots. Raw images of western blots [file 12943_2024_2115_MOESM10_ESM.pdf]
