# Supplementary material for: Pharmacological targeting of P300/CBP reveals EWS::FLI1-mediated senescence evasion in Ewing sarcoma
Source: Mol Cancer. 2024 Oct 5;23:222. doi: 10.1186/s12943-024-02115-7 (PMC11453018; doi:10.1186/s12943-024-02115-7)
Supplement: Supplementary file 8 — Supplementary Material 8: Figure S8: EWS::FLI1 and EWS::ERG bind to regulatory elements of senescence-related genes. (A) ChIP-Seq analysis explores EWS::FLI1 binding sites, H3K27ac and P300 in the regulatory elements of LMNB1, P15, P16, TP53 and P21 in SKNMC before and after EWS::FLI1 knockdown. (B) ChIP-Seq data shows EWS::FLI1 and acH3K27 binding sites in LMNB1, P15, P16, TP53 and P21 in MSC overexpressing EWS::FLI1. (C) ChIP-Seq analysis shows binding sites in the regulatory elements of LMNB1, P15, P16, TP53, and P21 for EWS::FLI1 in SKES1 and EWS::ERG in TC32. (D) Transcriptional levels (RNA-Seq) of P16, TP53 and P21 following EWS::FLI1 knockdown (96 hours). (E) P16, TP53 and P21 expression (RNA-Seq) in MSC overexpressing EWS::FLI1 and GFP control cells. Data are presented as mean expression levels ± SEM; p<0.05 by t-test (n=4). [file 12943_2024_2115_MOESM8_ESM.pptx]

## Slide 1
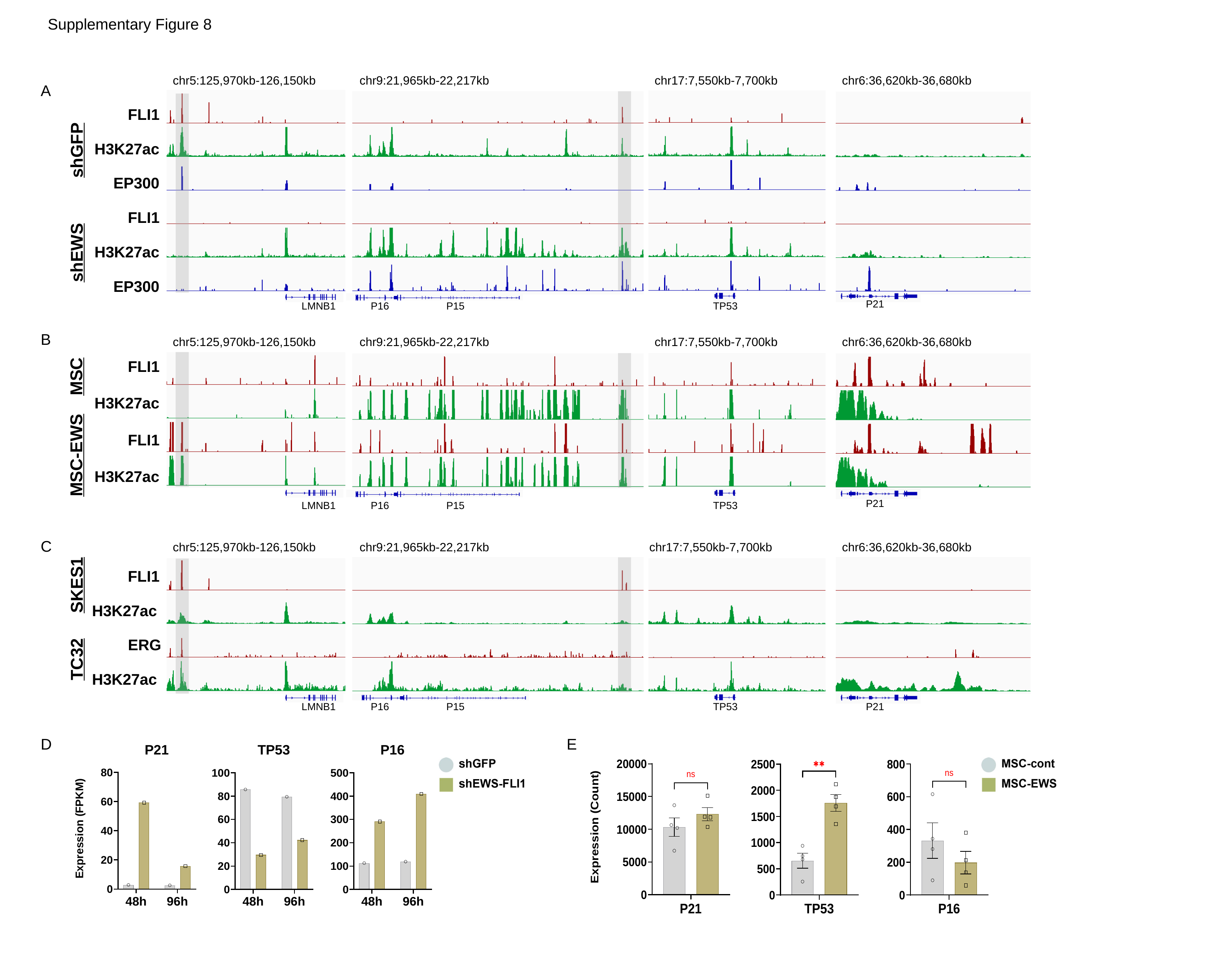

Supplementary Figure 8
chr5:125,970kb-126,150kb
chr9:21,965kb-22,217kb
chr17:7,550kb-7,700kb
chr6:36,620kb-36,680kb
A
FLI1
H3K27ac
shGFP
EP300
FLI1
H3K27ac
shEWS
EP300
P21
LMNB1
P16
P15
TP53
B
chr5:125,970kb-126,150kb
chr9:21,965kb-22,217kb
chr17:7,550kb-7,700kb
chr6:36,620kb-36,680kb
FLI1
MSC
H3K27ac
FLI1
MSC-EWS
H3K27ac
P21
LMNB1
P16
P15
TP53
C
chr5:125,970kb-126,150kb
chr9:21,965kb-22,217kb
chr17:7,550kb-7,700kb
chr6:36,620kb-36,680kb
FLI1
SKES1
H3K27ac
ERG
TC32
H3K27ac
LMNB1
P16
P15
TP53
P21
D
E
